# Comparison of efficacy and safety of drug-eluting versus uncoated balloon angioplasty for femoropopliteal arterial occlusive disease: a meta-analysis

**DOI:** 10.1186/s12872-020-01667-y

**Published:** 2020-08-31

**Authors:** Hai Feng, Xueming Chen, Xiaobo Guo, Zhe Zhang, Zhiwen Zhang, Bin Liu, Lishan Lian

**Affiliations:** Department of Vascular Surgery, Beijing Friendship Hospital, Capital Medical University, Beijing, 100050 China

**Keywords:** Drug-eluting balloon angioplasty, Uncoated balloon angioplasty, Femoropopliteal arterial occlusive disease, Meta-analysis, Randomized controlled trials

## Abstract

**Background:**

This quantitative meta-analysis was conducted to evaluate the efficacy and safety of drug-eluting balloon (DEB) vs. uncoated balloon (UCB) in patients with femoropopliteal arterial occlusive disease.

**Methods:**

Electronic databases were searched to identify randomized controlled trials (RCTs) that compared DEB and UCB till November 2018. The random-effects model was used for conducting pooled analyses.

**Results:**

Seventeen RCTs with 2706 patients were included in the final meta-analysis. Patients who received DEB had higher levels of minimal luminal diameter (MLD) at 6 (WMD: 0.77; 95%CI: 0.53 to 1.02; *P* < 0.001) and 12 months (WMD: 1.33; 95%CI: 0.93 to 1.73; *P* < 0.001) than those who received UCB. DEB reduced the late lumen loss (LLL) levels after 6 (WMD: -0.57; 95%CI: − 1.07 to − 0.06; *P* = 0.029) and 12 months (WMD: -0.95; 95%CI: − 1.28 to − 0.62; *P* < 0.001). DEB was found not superior over UCB on primary patency after 6 months (RR: 1.44; 95%CI: 0.88–2.35; *P* = 0.149), whereas DEB increased the primary patency after 12 (RR: 1.51; 95%CI: 1.25–1.83; *P* < 0.001) and 24 months (RR: 1.51; 95%CI: 1.30–1.77; *P* < 0.001). Patients who received DEB had reduced the risk of restenosis after 6 (RR: 0.47; 95%CI: 0.33–0.67; *P* < 0.001) and 12 months (RR: 0.55; 95%CI: 0.35–0.85; *P* = 0.008). DEB reduced the risk of major adverse events after 6 (RR: 0.30; 95%CI: 0.14–0.61; *P* = 0.001), 12 (RR: 0.49; 95%CI: 0.32–0.76; *P* = 0.001) and 24 months (RR: 0.62; 95%CI: 0.41–0.92; *P* = 0.018).

**Conclusions:**

DEB yielded additional benefits on MLD, LLL, primary patency, restenosis, TLR, and major adverse events than UCB in patients with femoropopliteal arterial occlusive disease.

## Background

Peripheral artery disease (PAD) is predominantly caused by atherosclerosis and manifested as an obstructive disease of major arteries. It always occurs in lower extremities, causing significant disability, limb loss, and mortality, especially in elderly population [1]. According to a study, there are more than 200 million individuals affected by PAD and is considered as a serious global health problem [2]. The common type of PAD is femoropopliteal arterial occlusive disease, and is mainly managed by intermittent claudication and severe limb ischemia, lowering the quality of life of patients [3]. Currently, the treatment strategies for PAD included surgical approaches, conservative treatments, exercise training, or endovascular techniques, and these techniques are widely used for the treatment of femoropopliteal arterial occlusive disease [4].

Currently, a drug-eluting balloon (DEB) using paclitaxel that is homogeneously coated on the balloon surface is recommended for PAD, which is then subsequently released into the lesion upon contact with the vessel wall. Moreover, it could improve the blood flow rate and reduce restenosis than drug-coated stents [5]. A previous meta-analysis study found that DEB was associated with increased durability of treatment effect, improved binary restenosis, late lumen loss (LLL), and target lesion revascularization (TLR) after short- and mid-term follow-up [6]. However, the analysis was conducted based on just nine studies and whether the treatment effects of DEB versus uncoated balloon (UCB) for femoropopliteal arterial occlusive disease are different according to the patients’ characteristics are not illustrated. Therefore, the current quantitative meta-analysis was conducted to compare the efficacy and safety of DEB with UCB in patients with femoropopliteal arterial occlusive disease.

## Methods

This was a meta-analysis study of randomized controlled trials (RCTs), and so IRB approval was not required.

### Data sources, search strategy, and selection criteria

This systematic review and meta-analysis was conducted according to the Preferred Reporting Items for Systematic Reviews and Meta-Analysis (PRISMA) Statement issued in 2009 [7]. RCTs published in English language and those that investigated the treatment effects of DEB versus UCB in patients with femoropopliteal arterial occlusive disease were considered eligible in this meta-analysis. We systematically searched PubMed, Embase, and the Cochrane library for studies throughout November 2018. The medical subject headings and free words such as (“drug-eluting balloon” or “coated balloon”) and (“femoral” or “femoral artery” or “femoropopliteal” or “infrainguinal”) were searched. Moreover, the registered and unpublished RCTs were also reviewed from the website http://clinicaltrials.gov/ (US, NIH). Finally, the reference lists of the retrieved studies were manually searched to check if there are any new eligible trials.

Two authors conducted the literature search and study selection was done by following a standardized approach. Any disagreement between the authors was settled by group discussion until a mutual consensus was reached. The studies should meet the following inclusion criteria: (1) Patients: all patients are diagnosed with femoropopliteal arterial occlusive disease; (2) Intervention: DEB; (3) Control: UCB; (4) Outcomes: minimal luminal diameter (MLD), LLL, primary patency, restenosis, TLR, all-cause mortality, major adverse events, target lesion thrombosis, and amputation; and (5) Study design: the studies should have RCT design. The exclusion criteria were as follows: (1) patients diagnosed with other PADs; (2) studies with observational study design; (3) studies without appropriate control; and (4) unavailability of investigated outcomes.

### Data collection and quality assessment

Two authors extracted the data from the eligible trials based on the standardized protocol. The collected data included the study group’s name, publication year, country, sample size, mean age, percent male, smoker, diabetes mellitus (DM), hyperlipidemia, and hypertension, baseline ankle-brachial index (ABI), mean lesion, intervention, control, follow-up duration, and reported outcomes. The quality of retrieved studies was evaluated by JADAD scale, which is based on randomization, blinding, allocation concealment, withdrawals and dropouts, and the use of intention-to-treat analysis [8]. The quality assessment was assessed by two authors, and any inconsistencies were resolved by an additional author by referring to the original article.

### Statistical analysis

The results of MLD and LLL are presented as continuous data, and weighted mean difference (WMD) and 95% confidence interval (CI) in each trial are calculated based on mean, standard deviation, and sample size in DEB and UCB groups. Moreover, the incidence of primary patency, restenosis, TLR, all-cause mortality, major adverse events, target lesion thrombosis, and amputation are defined as categorical data, and relative risk (RR) with 95%CI were calculated based on the events that occurred and sample size in DEB and UCB groups. The pooled WMD and RR for investigated outcomes were analyzed using the random-effects model [9, 10]. The I-square and Q statistic were employed for evaluating heterogeneity, and *P* < 0.10 indicates significant heterogeneity [11, 12]. For investigating the stability of pooled results, greater than five studies were evaluated using sensitivity analysis [13]. Moreover, subgroup analyses were conducted based on mean age, smoker percent, DM percent, hyperlipidemia percent, hypertension percent, and paclitaxel dose. Univariable meta-regression analyses were also conducted to explore the impact of these factors [14]. For analyzing publication bias of outcomes, funnel plots, Egger [15], and Begg [16] tests were used for assessing the results of more than 5 studies. *P*-values of pooled results are 2-sided, and *P* < 0.05 was considered to be statistically significant. The analyses of this study were conducted by STATA software (Version 10.0; StataCorp, Texas, United States of America).

## Results

### Literature search

**Fig. 1:**
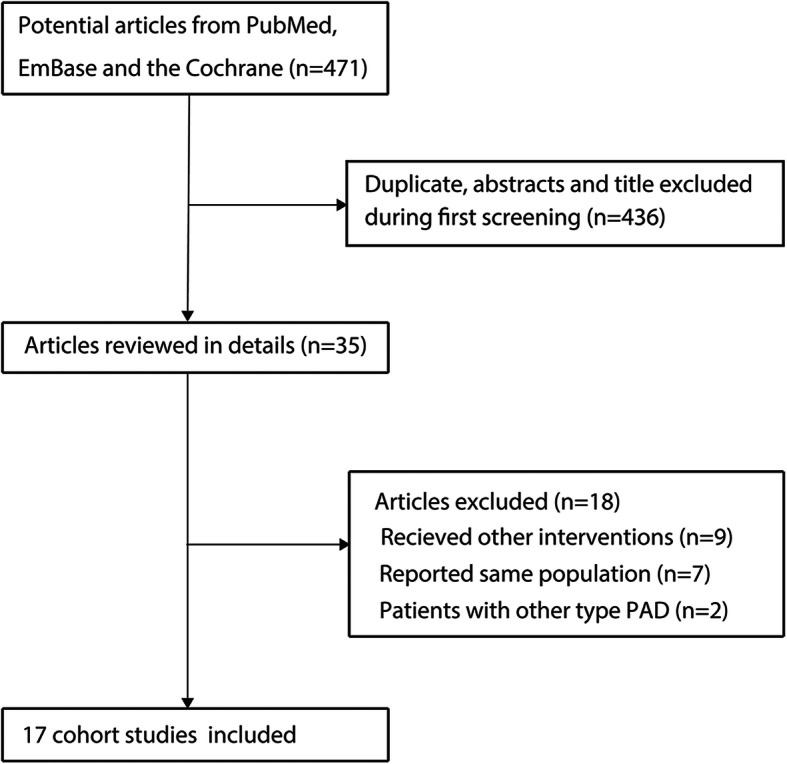
Flow diagram of literature search and trial selection process

The initial electronic search produced 471 articles, and 436 of these were discarded due to duplication and irrelevant topics. The remaining 35 studies were retrieved for full-text evaluation. Of these, 18 studies were excluded due to the following reasons: other interventions (*n* = 9), trials reporting the same populations (*n* = 7), and patients with other PAD (*n* = 2). Finally, 17 RCTs were considered eligible for final meta-analysis [17–33]. Review of the reference lists of these studies yielded no new eligible study. The details of literature search and selection process are presented in Fig. 1.

### Study characteristics

The baseline characteristics of included studies and patients are summarized in Table 1. Overall, 17 RCTs, including a total of 2706 patients, were eligible in this study. The reported outcomes at 6, 12, and 24 months were abstracted, and 50–476 patients were included in each trial. The mean age of included patients ranged from 65.8–75.0 years, and the percent male ranged from 56.7–79.2%. The percent of smokers ranged from 31.0–86.3%, and percent DM ranged from 25.0–100.0%. The percent of hyperlipidemia ranged from 28.0–90.0%, and percent of hypertension ranged from 65.9–93.7%. Nine RCTs had a JADAD score of 4, and the remaining 8 RCTs had a score of 3.

**Table 1:** The baseline characteristics of included studies and recruited patients

Study	Country	Sample size	Mean age (years)	Men (%)	Smoker (%)	DM (%)	Hyperlipidemia (%)	Hypertension (%)	ABI	Mean lesion (cm)	Intervention and control	Follow-up duration	Study quality
AcoArt I 2016 [17]	China	200	65.8	73.5	31.0	55.5	28.0	67.0	0.46	14.9	Paclitaxel-coated (3.0 μg/mm^2^) and standard uncoated balloon catheters	6 and 12 months	4
BIOLUX P-I 2015 [18]	Germany	60	70.8	56.7	68.8	33.4	61.7	73.4	0.70	6.0	Paclitaxel-coated (3.0 μg/mm^2^) balloon or the uncoated balloon	6 and 12 months	3
BIOLUX P-II 2015 [19]	Germany	72	71.3	79.2	55.6	66.7	68.1	86.1	NA	NA	Passeo-18 Lux paclitaxel-coated (3.0 μg/mm^2^) drug-eluting balloon or Passeo-18 percutaneous transluminal angioplasty	6 and 12 months	3
CONSEQUENT 2017 [20]	Germany	153	68.1	68.0	47.7	36.6	54.4	78.4	NA	13.2	Paclitaxel-coated (3.0 μg/mm^2^) balloons or plain old balloon angioplasty	6, 12, and 24 months	4
DEBATE-SFA 2013 [21]	Italy	104	75.0	69.2	51.0	74.0	57.7	88.5	0.32	9.5	Paclitaxel-eluting (3.0 μg/mm^2^) balloon plus bare-metal stent or percutaneous transluminal angioplasty plus bare-metal stent	12 months	4
DEBELLUM 2012 [22]	Italy	50	67.0	74.0	62.0	44.0	58.0	68.0	0.56	7.5	Paclitaxel-eluting (3.5 μg/mm^2^) balloon or angioplasty balloon	6 and 12 months	4
ILLUMENATE Pivotal 2017 [23]	US, Austria	300	68.8	58.7	81.0	50.3	88.7	93.7	0.75	8.3	Stellarex Drug-Coated (2.0 μg/mm^2^) Balloon or percutaneous transluminal angioplasty	12 months	3
ILLUMENATE European 2017 [24]	Germany, Austria	299	66.4	69.9	86.3	36.5	62.2	77.9	0.71	7.2	Low-dose paclitaxel coated (2.0 μg/mm^2^) balloon or percutaneous transluminal angioplasty	12 months	3
IN.PACT SFA 2015 [25]	US, Europe	331	67.7	65.9	37.8	43.2	83.7	90.3	0.76	8.9	Paclitaxel-coated (3.5 μg/mm^2^) balloons or percutaneous transluminal angioplasty	12 and 24 months	4
IN.PACT 2017 [26]	Belgium	106	70.5	65.1	57.5	100.0	73.6	88.7	NA	7.6	Paclitaxel-coated (3.5 μg/mm^2^) balloons or plain old balloon angioplasty	6 months	3
ISAR-STATH 2017 [27]	Germany	100	69.4	70.0	70.0	25.0	90.0	80.0	0.75	7.1	Paclitaxel-coated (3.5 μg/mm^2^) balloons or plain balloon angioplasty	6 and 24 months	3
LEVANT I2014 [28]	Germany	101	68.5	67.3	34.7	47.5	64.4	91.1	0.64	8.0	Lutonix paclitaxel-coated (2.0 μg/mm^2^) balloons or uncoated balloons	6, 12, and 24 months	4
LEVANT 22015 [29]	US, Europe	476	68.2	63.0	34.7	42.9	88.4	88.7	0.74	NA	Paclitaxel-coated (2.0 μg/mm^2^) balloon or standard angioplasty	12 months	4
PACIFIER 2012 [30]	Germany	91	71.0	61.5	53.8	35.2	48.4	65.9	0.69	6.8	Paclitaxel-coated (3.5 μg/mm^2^) IN.PACT Pacific or uncoated Pacific balloons	6 and 12 months	4
PACUBA 2016 [31]	Austria	74	68.2	58.1	47.3	40.5	58.1	71.6	0.65	17.9	Paclitaxel-based drug-eluting (3.0 μg/mm^2^) balloon angioplasty or standard percutaneous transluminal angioplasty	6 and 12 months	4
THUNDER 2008 [32]	Germany	102	68.5	63.7	22.5	48.0	65.7	81.4	0.50	7.4	Paclitaxel-coated (3.0 μg/mm^2^) balloons or uncoated balloons	6, 12, and 24 months	3
FemPac 2008 [33]	Germany	87	68.7	59.8	41.4	47.1	57.5	79.3	0.70	4.3	Paclitaxel-coated (3.0 μg/mm^2^) balloons or uncoated balloons	6 and 24 months	3

*ABI* Ankle-brachial index, *DM* Diabetes mellitus

### MLD

**Fig. 2:**
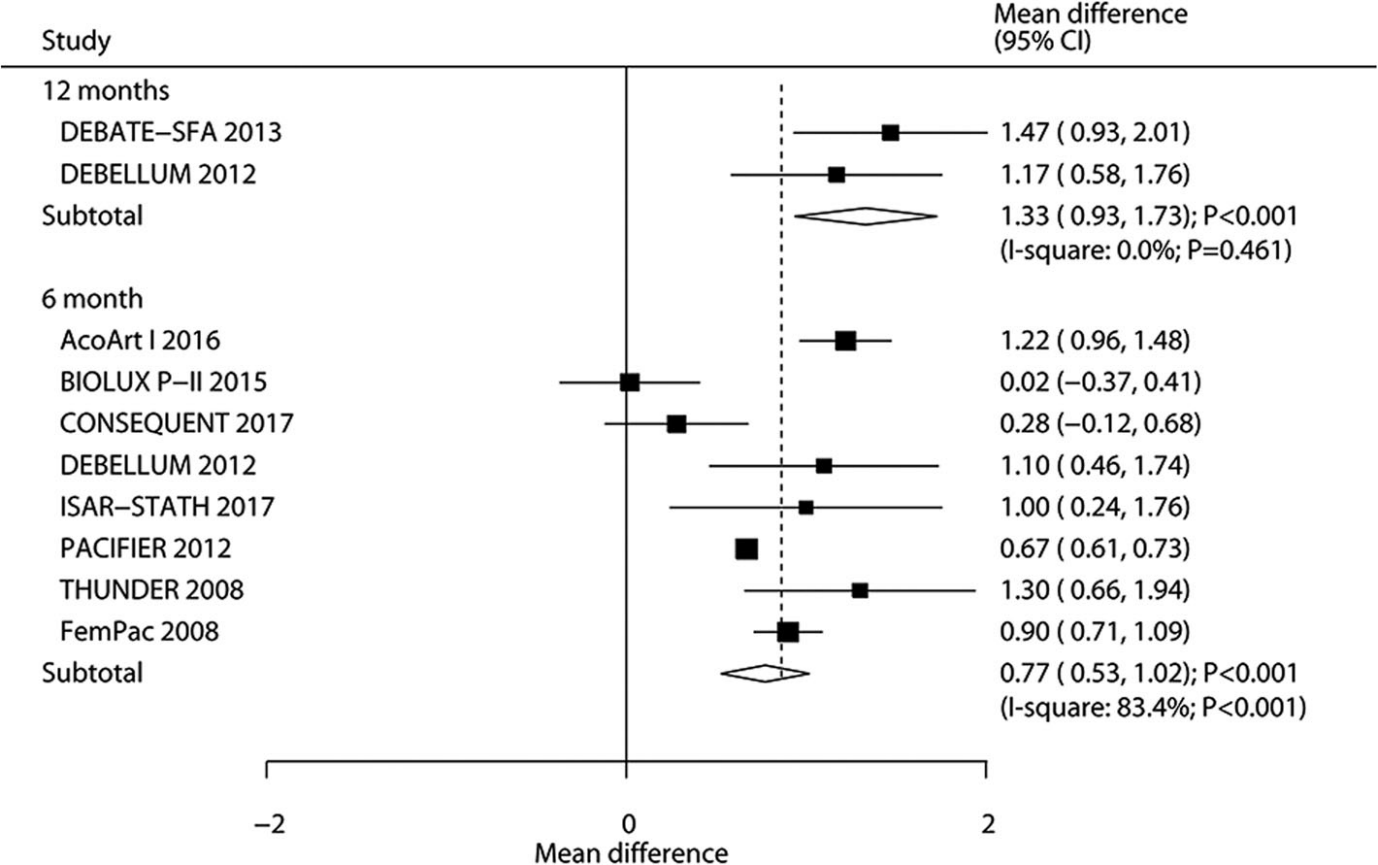
DEB versus UCB on MLD

The breakdown of the number of trials that reported MLD after 6 and 12 months were 8 and 2 trials, respectively. The summary WMD indicated that patients who received DEB showed association with higher levels of MLD at 6 months (WMD: 0.77; 95%CI: 0.53 to 1.02; *P* < 0.001) and 12 months (WMD: 1.33; 95%CI: 0.93 to 1.73; *P* < 0.001) than those who received UCB (Fig. 2). There was significant heterogeneity for MLD at 6 months, whereas no evidence of heterogeneity for MLD at 12 months. Sensitivity analysis results indicated that the pooled conclusion was stable and did not alter by excluding any particular trial (Supplemental 1). Univariable meta-regression indicated that mean age (*P* < 0.001), percent of smoker (*P* < 0.001), and paclitaxel dose (*P* = 0.032) could affect the treatment of DEB versus UCB on MLD after 6 months (Table 2). Subgroup analysis indicated significant differences of DEB versus UCB on MLD at 6 months in most of the subsets, whereas no significant difference was observed when mean age was ≥70.0 years, DM percent ≥50.0%, hyperlipidemia percent ≥60.0%, and hypertension ≥80.0% (Table 2). No significant publication bias for MLD at 6 months was observed (*P*-value for Egger: 0.569; *P* value for Begg: 0.902; Supplemental 2).

**Table 2:** Subgroup analyses for investigated outcomes

Outcomes	Subgroup	WMD or RR and 95% CI	*P* value	Heterogeneity (%)	*P* value for Meta-regression
MLD at 6 months	Mean age (years)				
	≥ 70.0	0.37 (− 0.26 to 1.01)	0.247	90.4 (0.001)	< 0.001
	< 70.0	0.94 (0.64 to 1.24)	< 0.001	70.0 (0.005)	
	Smoker (%)				
	≥ 50.0	0.63 (0.21 to 1.05)	0.003	76.9 (0.005)	< 0.001
	< 50.0	0.91 (0.53 to 1.29)	< 0.001	81.7 (0.001)	
	DM (%)				
	≥ 50.0	0.63 (−0.55 to 1.81)	0.294	96.0 (< 0.001)	0.140
	< 50.0	0.78 (0.57 to 1.00)	< 0.001	66.1 (0.011)	
	Hyperlipidemia (%)				
	≥ 60.0	0.74 (−0.14 to 1.61)	0.099	85.0 (0.001)	0.127
	< 60.0	0.82 (0.56 to 1.09)	< 0.001	84.9 (< 0.001)	
	Hypertension (%)				
	≥ 80.0	0.74 (−0.14 to 1.61)	0.099	85.0 (0.001)	0.127
	< 80.0	0.82 (0.56 to 1.09)	< 0.001	84.9 (< 0.001)	
	Dose of paclitaxel				
	3.0 μg/mm^2^	0.73 (0.30 to 1.17)	0.001	88.6 (< 0.001)	0.032
	3.5 μg/mm^2^	0.74 (0.52 to 0.95)	< 0.001	17.9 (0.296)	
LLL at 6 months	Mean age (years)				
	≥ 70.0	0.08 (−0.61 to 0.78)	0.815	94.8 (< 0.001)	< 0.001
	< 70.0	−0.83 (−1.14 to − 0.52)	< 0001	90.3 (< 0.001)	
	Smoker (%)				
	≥ 50.0	−0.37 (− 1.10 to 0.36)	0.320	95.4 (< 0.001)	< 0.001
	< 50.0	−0.75 (−1.08 to − 0.41)	< 0.001	93.2 (< 0.001)	
	DM percent (%)				
	≥ 50.0	−0.55 (−1.64 to 0.55)	0.329	96.7 (< 0.001)	< 0.001
	< 50.0	−0.57 (−1.14 to − 0.00)	0.049	99.0 (< 0.001)	
	Hyperlipidemia (%)				
	≥ 60.0	−0.72 (−1.37 to − 0.07)	0.031	86.1 (< 0.001)	< 0.001
	< 60.0	−0.44 (−1.12 to 0.24)	0.201	99.4 (< 0.001)	
	Hypertension (%)				
	≥ 80.0	−0.80 (−1.76 to 016)	0.102	90.7 (< 0.001)	< 0.001
	< 80.0	−0.46 (−1.08 to 0.16)	0.148	99.3 (< 0.001)	
	Dose of paclitaxel				
	3.0 μg/mm^2^	−0.59 (−0.87 to − 0.31)	< 0.001	90.5 (< 0.001)	< 0.001
	3.5 μg/mm^2^	− 0.51 (−1.99 to 0.97)	0.500	96.0 (< 0.001)	
Primary patency at 12 months	Mean age (years)				
	≥ 70.0	–	–	–	–
	< 70.0	1.51 (1.25–1.83)	< 0.001	67.8 (0.008)	
	Smoker (%)				
	≥ 50.0	1.37 (1.15–1.63)	< 0.001	–	0.445
	< 50.0	1.58 (1.22–2.03)	< 0.001	73.3 (0.005)	
	DM (%)				
	≥ 50.0	2.26 (1.65–3.09)	< 0.001	–	0.003
	< 50.0	1.38 (1.20–1.59)	< 0.001	39.5 (0.158)	
	Hyperlipidemia (%)				
	≥ 60.0	1.36 (1.22–1.52)	< 0.001	12.7 (0.329)	0.001
	< 60.0	2.34 (1.74–3.15)	< 0.001	0.0 (0.506)	
	Hypertension (%)				
	≥ 80.0	1.35 (1.14–1.61)	0.001	41.8 (0.180)	0.254
	< 80.0	1.92 (1.18–3.11)	0.008	81.5 (0.004)	
	Dose of paclitaxel				
	2.0 μg/mm^2^	1.29 (1.15–1.46)	< 0.001	0.0 (0.696)	0.001
	3.0 μg/mm^2^	2.34 (1.74–3.15)	< 0.001	0.0 (0.506)	
	3.5 μg/mm^2^	1.57 (1.29–1.91)	< 0.001	–	
Restenosis at 6 months	Mean age (years)				
	≥ 70.0	0.58 (0.28–1.19)	0.137	71.4 (0.015)	0.005
	< 70.0	0.38 (0.29–0.49)	< 0.001	0.0 (0.850)	
	Smoker percent (%)				
	≥ 50.0	0.56 (0.31–0.99)	0.047	66.1 (0.019)	0.009
	< 50.0	0.37 (0.28–0.49)	< 0.001	0.0 (0.770)	
	DM (%)				
	≥ 50.0	0.61 (0.27–1.40)	0.242	88.4 (< 0.001)	0.200
	< 50.0	0.41 (0.29–0.56)	< 0.001	0.0 (0.973)	
	Hyperlipidemia (%)				
	≥ 60.0	0.58 (0.34–0.97)	0.038	64.5 (0.024)	0.008
	< 60.0	0.36 (0.26–0.48)	< 0.001	0.0 (0.715)	
	Hypertension (%)				
	≥ 80.0	0.62 (0.35–1.09)	0.098	70.0 (0.019)	0.004
	< 80.0	0.36 (0.27–0.48)	< 0.001	0.0 (0.849)	
	Dose of paclitaxel				
	3.0 μg/mm^2^	0.48 (0.29–0.81)	0.005	72.7 (0.003)	1.000
	3.5 μg/mm^2^	0.48 (0.32–0.71)	< 0.001	0.0 (0.506)	
TLR at 6 months	Mean age (years)				
	≥ 70.0	0.56 (0.31–1.00)	0.051	0.0 (0.522)	0.075
	< 70.0	0.31 (0.18–0.52)	< 0.001	42.9 (0.105)	
	Smoker (%)				
	≥ 50.0	0.57 (0.33–0.98)	0.041	0.0 (0.720)	0.035
	< 50.0	0.28 (0.16–0.50)	< 0.001	45.5 (0.102)	
	DM (%)				
	≥ 50.0	0.32 (0.08–1.36)	0.124	86.2 (0.007)	1.000
	< 50.0	0.37 (0.24–0.56)	< 0.001	10.0 (0.353)	
	Hyperlipidemia (%)				
	≥ 60.0	0.49 (0.26–0.92)	0026	31.2 (0.213)	0.041
	< 60.0	0.27 (0.17–0.45)	< 0.001	19.8 (0.289)	
	Hypertension (%)				
	≥ 80.0	0.46 (0.23–0.94)	0.033	46.5 (0.133)	0.062
	< 80.0	0.28 (0.17–0.46)	< 0.001	14.9 (0.319)	
	Dose of paclitaxel				
	2.0 μg/mm^2^	0.57 (0.23–1.45)	0.241	–	0.039
	3.0 μg/mm^2^	0.26 (0.15–0.45)	< 0.001	31.5 (0.199)	
	3.5 μg/mm^2^	0.56 (0.32–0.97)	0.037	0.0 (0.558)	
TLR at 12 months	Mean age (years)				
	≥ 70.0	0.53 (0.21–1.35)	0.183	63.7 (0.064)	0.421
	< 70.0	0.42 (0.29–1.60)	< 0.001	69.9 (< 0.001)	
	Smoker (%)				
	≥ 50.0	0.47 (0.31–0.72)	< 0.001	36.6 (0.163)	1.000
	< 50.0	0.41 (0.25–0.67)	< 0.001	79.0 (< 0.001)	
	DM (%)				
	≥ 50.0	0.48 (0.18–1.30)	0.149	83.5 (0.002)	0.778
	< 50.0	0.43 (0.30–0.61)	< 0.001	62.3 (0.005)	
	Hyperlipidemia (%)				
	≥ 60.0	0.47 (0.30–0.75)	0.001	69.5 (0.002)	1.000
	< 60.0	0.38 (0.22–0.65)	< 0.001	70.1 (0.010)	
	Hypertension (%)				
	≥ 80.0	0.50 (0.28–0.89)	0.018	76.5 (0.001)	0.297
	< 80.0	0.39 (0.26–0.58)	< 0.001	56.1 (0.034)	
	Dose of paclitaxel				
	2.0 μg/mm^2^	0.62 (0.43–0.88)	0.007	26.1 (0.255)	0.007
	3.0 μg/mm^2^	0.44 (0.26–0.76)	0.003	74.7 (0.001)	
	3.5 μg/mm^2^	0.24 (0.13–0.42)	< 0.001	14.2 (0.312)	
TLR at 24 months	Mean age (years)				
	≥ 70.0	–	–	–	–
	< 70.0	0.42 (0.30–0.58)	< 0.001	35.6 (0.169)	
	Smoker (%)				
	≥ 50.0	0.45 (0.20–0.98)	0.045	–	1.000
	< 50.0	0.41 (0.28–0.60)	< 0.001	48.6 (0100)	
	DM (%)				
	≥ 50.0	–	–	–	–
	< 50.0	0.42 (0.30–0.58)	< 0.001	35.6 (0.169)	
	Hyperlipidemia (%)				
	≥ 60.0	0.43 (0.28–0.68)	< 0.001	51.9 (0.100)	0.624
	< 60.0	0.38 (0.22–0.65)	< 0.001	22.2 (0.257)	
	Hypertension (%)				
	≥ 80.0	0.43 (0.28–0.68)	< 0.001	51.9 (0.100)	0.624
	< 80.0	0.38 (0.22–0.65)	< 0.001	22.2 (0.257)	
	Dose of paclitaxel				
	2.0 μg/mm^2^	0.73 (0.44–1.22)	0.233	–	0.059
	3.0 μg/mm^2^	0.35 (0.24–0.52)	< 0.001	0.0 (0.401)	
	3.5 μg/mm^2^	0.37 (0.24–0.57)	< 0.001	0.0 (0.593)	
All-cause mortality at 6 months	Mean age (years)				
	≥ 70.0	0.65 (0.18–2.29)	0.499	0.0 (0.567)	0.481
	< 70.0	1.26 (0.34–4.69)	0.732	0.0 (0.517)	
	Smoker (%)				
	≥ 50.0	0.81 (0.25–2.61)	0.719	0.0 (0.577)	0.797
	< 50.0	1.03 (0.24–4.39)	0.963	0.0 (0.393)	
	DM percent (%)				
	≥ 50.0	1.04 (0.22–4.91)	0.962	–	0.803
	< 50.0	0.82 (0.27–2.53)	0.732	0.0 (0.571)	
	Hyperlipidemia (%)				
	≥ 60.0	0.94 (0.34–2.56)	0.900	0.0 (0.655)	0.804
	< 60.0	0.71 (0.05–9.49)	0.797	28.9 (0.236)	
	Hypertension (%)				
	≥ 80.0	1.05 (0.37–3.03)	0.924	0.0 (0.576)	0.536
	< 80.0	0.05 (0.09–3.31)	0.513	0.0 (0.462)	
	Dose of paclitaxel				
	2.0 μg/mm^2^	0.32 (0.03–2.96)	0.315	–	0.564
	3.0 μg/mm^2^	1.44 (0.28–7.30)	0.663	0.0 (0.567)	
	3.5 μg/mm^2^	0.93 (0.26–3.30)	0.910	0.0 (0.443)	
All-cause mortality at 12 months	Mean age (years)				
	≥ 70.0	0.54 (0.10–2.93)	0.474	30.4 (0.238)	0.815
	< 70.0	0.94 (0.47–1.89)	0.860	0.0 (0.771)	
	Smoker (%)				
	≥ 50.0	0.83 (0.28–2.46)	0.741	7.1 (0.357)	1.000
	< 50.0	0.88 (0.41–1.91)	0.746	0.0 (0.663)	
	DM (%)				
	≥ 50.0	1.13 (0.42–3.02)	0.806	0.0 (0.749)	0503
	< 50.0	0.74 (0.33–1.63)	0.450	0.0 (0.488)	
	Hyperlipidemia (%)				
	≥ 60.0	0.94 (0.47–1.92)	0.873	0.0 (0.627)	0.686
	< 60.0	0.67 (0.19–2.42)	0.546	0.0 (0.401)	
	Hypertension (%)				
	≥ 80.0	1.04 (0.50–2.14)	0.926	0.0 (0.663)	0.403
	< 80.0	0.56 (0.17–1.81)	0.332	0.0 (0.500)	
	Dose of paclitaxel				
	2.0 μg/mm^2^	0.82 (0.35–1.88)	0.632	0.0 (0.698)	0.973
	3.0 μg/mm^2^	0.98 (0.35–2.73)	0.965	0.0 (0.575)	
	3.5 μg/mm^2^	0.83 (0.03–24.78)	0.915	63.0 (0.100)	
Major adverse events at 12 months	Mean age (years)				
	≥ 70.0	0.52 (0.20–1.36)	0.183	71.9 (0.029)	0.196
	< 70.0	0.44 (0.29–0.66)	< 0.001	0.0 (0.694)	
	Smoker (%)				
	≥ 50.0	0.49 (0.32–0.76)	0.001	47.5 (0.090)	–
	< 50.0	–	–	–	
	DM (%)				
	≥ 50.0	0.75 (0.38–1.46)	0.400	59.6 (0.116)	0.018
	< 50.0	0.37 (0.24–0.57)	< 0.001	0.0 (0.685)	
	Hyperlipidemia (%)				
	≥ 60.0	0.59 (0.36–0.95)	0.031	46.2 (0.134)	0.083
	< 60.0	0.33 (0.17–0.62)	0.001	0.0 (0.333)	
	Hypertension (%)				
	≥ 80.0	0.75 (0.38–1.46)	0.400	59.6 (0.116)	0.018
	< 80.0	0.37 (0.24–0.57)	< 0.001	0.0 (0.685)	
	Dose of paclitaxel				
	2.0 μg/mm^2^	0.45 (0.28–0.74)	0.001	0.0 (0.418)	0.046
	3.0 μg/mm^2^	0.79 (0.39–1.60)	0.514	43.5 (0.184)	
	3.5 μg/mm^2^	0.33 (0.17–0.62)	0.001	0.0 (0.333)	
Amputation at 12 months	Mean age (years)				
	≥ 70.0	0.56 (0.05–5.91)	0.632	–	0.857
	< 70.0	0.72 (0.20–2.61)	0.616	0.0 (0.802)	
	Smoker (%)				
	≥ 50.0	0.50 (0.12–2.11)	0.348	0.0 (0.855)	0.506
	< 50.0	1.11 (0.18–6.99)	0.910	0.0 (0.633)	
	DM (%)				
	≥ 50.0	0.47 (0.07–3.09)	0.429	0.0 (0.792)	0.627
	< 50.0	0.84 (0.20–3.43)	0.805	0.0 (0.714)	
	Hyperlipidemia (%)				
	≥ 60.0	1.08 (0.25–4.58)	0.922	0.0 (0.877)	0.321
	< 60.0	0.33 (0.05–2.03)	0.232	0.0 (0.996)	
	Hypertension (%)				
	≥ 80.0	1.10 (0.22–5.60)	0.907	0.0 (0.712)	0.419
	< 80.0	0.43 (0.09–2.08)	0.296	0.0 (0.845)	
	Dose of paclitaxel				
	2.0 μg/mm^2^	1.60 (0.25–10.06)	0.617	0.0 (0.899)	0.500
	3.0 μg/mm^2^	0.47 (0.07–3.09)	0.429	0.0 (0.792)	
	3.5 μg/mm^2^	0.33 (0.04–2.99)	0.326	–	

### LLL

**Fig. 3:**
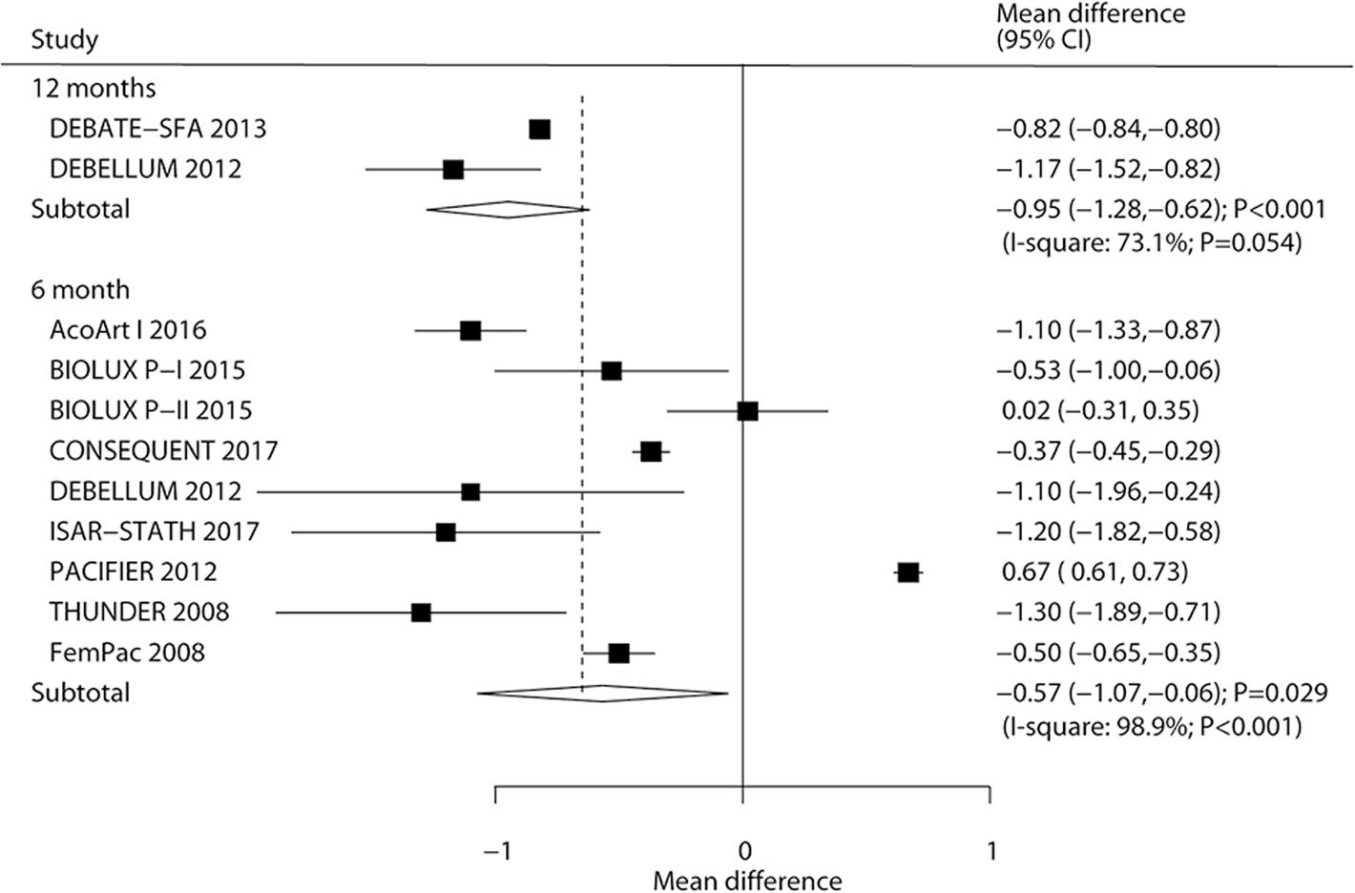
DEB versus UCB on LLL

The number of trials that reported LLL after 6 months and 12 months was nine trials and two trials, respectively. DEB significantly reduced LLL levels after 6 months (WMD: -0.57; 95%CI: − 1.07 to − 0.06; *P* = 0.029) and 12 months (WMD: -0.95; 95%CI: − 1.28 to − 0.62; *P* < 0.001) follow-up duration (Fig. 3), and substantial heterogeneity was observed across the included studies. Sensitivity analysis indicated that the LLL level after 6 months varied due to marginal 95%CI (Supplemental 1). The results of regression analysis indicated that mean age (*P* < 0.001), smoker percent (*P* < 0.001), DM percent (*P* < 0.001), hyperlipidemia percent (*P* < 0.001), hypertension percent (*P* < 0.001), and paclitaxel dose (*P* < 0.001) could bias the treatment effect of DEB versus UCB on LLL level (Table 2). Subgroup analysis indicated that DEB was associated with lower LLL if the mean age of the patients was < 70.0 years, smoker percent was < 50.0%, DM percent was < 50.0%, hyperlipidemia percent was ≥60.0%, and treated with 3.0 μg/mm^2^of paclitaxel. There was no evidence of publication bias for LLL after 6 months (*P*-value for Egger: 0.142; *P* value for Begg: 0.754; Supplemental 2).

### Primary patency

**Fig. 4:**
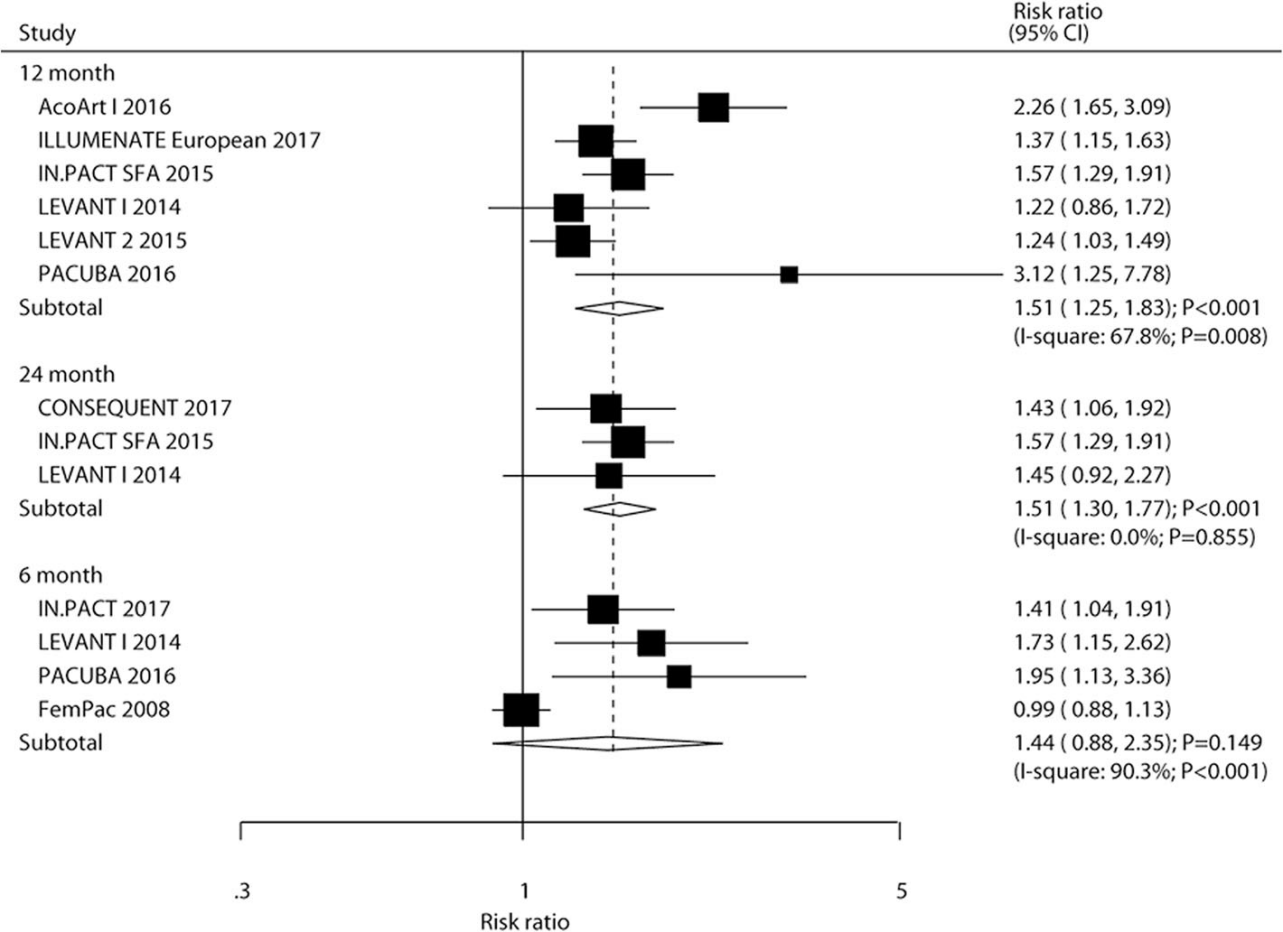
DEB versus UCB on the risk of primary patency

The number of trials that reported primary patency after 6, 12, and 24 months was four, six, and three, respectively. The summary RR indicated that DEB was not superior to UCB on primary patency after 6 months (RR: 1.44; 95%CI: 0.88–2.35; *P* = 0.149), whereas DEB significantly increased the primary patency after 12 months (RR: 1.51; 95%CI: 1.25–1.83; *P* < 0.001) and 24 months (RR: 1.51; 95%CI: 1.30–1.77; *P* < 0.001) when compared with UCB (Fig. 4). The summary conclusion for primary patency after 12 months remained unchanged after sequential exclusion of individual trials (Supplemental 1). DM percent (*P* = 0.003), hyperlipidemia percent (*P* = 0.001), and paclitaxel dose (*P* = 0.001) could affect the efficacy of DEB and primary patency (Table 2). However, the incidence of primary patency after 12 months in patients who received DEB versus UCB was persistent with statistical significance in all the subsets. No significant publication bias for primary patency after 12 months was observed (*P*-value for Egger: 0.238; *P* value for Begg: 0.452; Supplemental 2).

### Restenosis

**Fig. 5:**
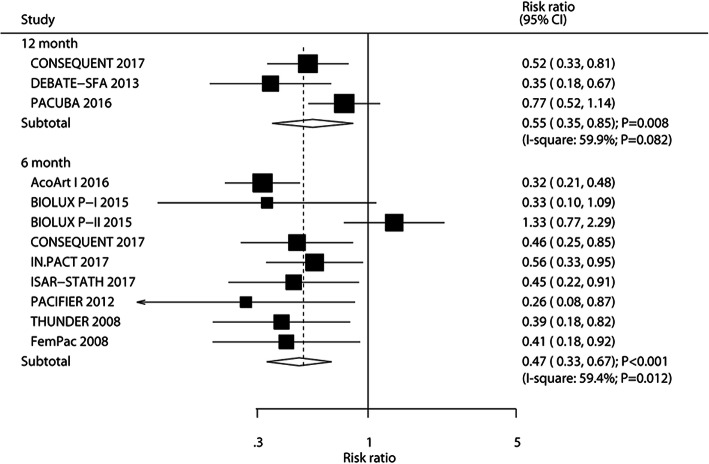
DEB versus UCB on the risk of restenosis

The number of trials that reported primary patency after 6 and 12 months was nine and three trials, respectively. We noted that patients who received DEB were associated with reduced risk of restenosis after 6 months (RR: 0.47; 95%CI: 0.33–0.67; *P* < 0.001) and 12 months (RR: 0.55; 95%CI: 0.35–0.85; *P* = 0.008), (Fig. 5). A significant potential heterogeneity was observed among the included trials. The results of sensitivity analysis indicated that the risk of restenosis after 6 months was stable and unchanged by removing any individual trial (Supplemental 1). Meta-regression analyses indicated that mean age (*P* = 0.005), smoker percent (*P* = 0.009), hyperlipidemia percent (*P* = 0.008), and hypertension percent (*P* = 0.004) might bias the effect of DEB and restenosis after 6 months (Table 2). No significant difference between DEB and UCB was found for the risk of restenosis after 6 months when the mean age of patients was ≥70.0 years, DM percent was ≥50.0%, and hypertension percent was ≥80.0%. No significant publication bias for restenosis after 6 months was observed (*P*-value for Egger: 0.741; *P* value for Begg: 0.175; Supplemental 2).

### TLR

**Fig. 6:**
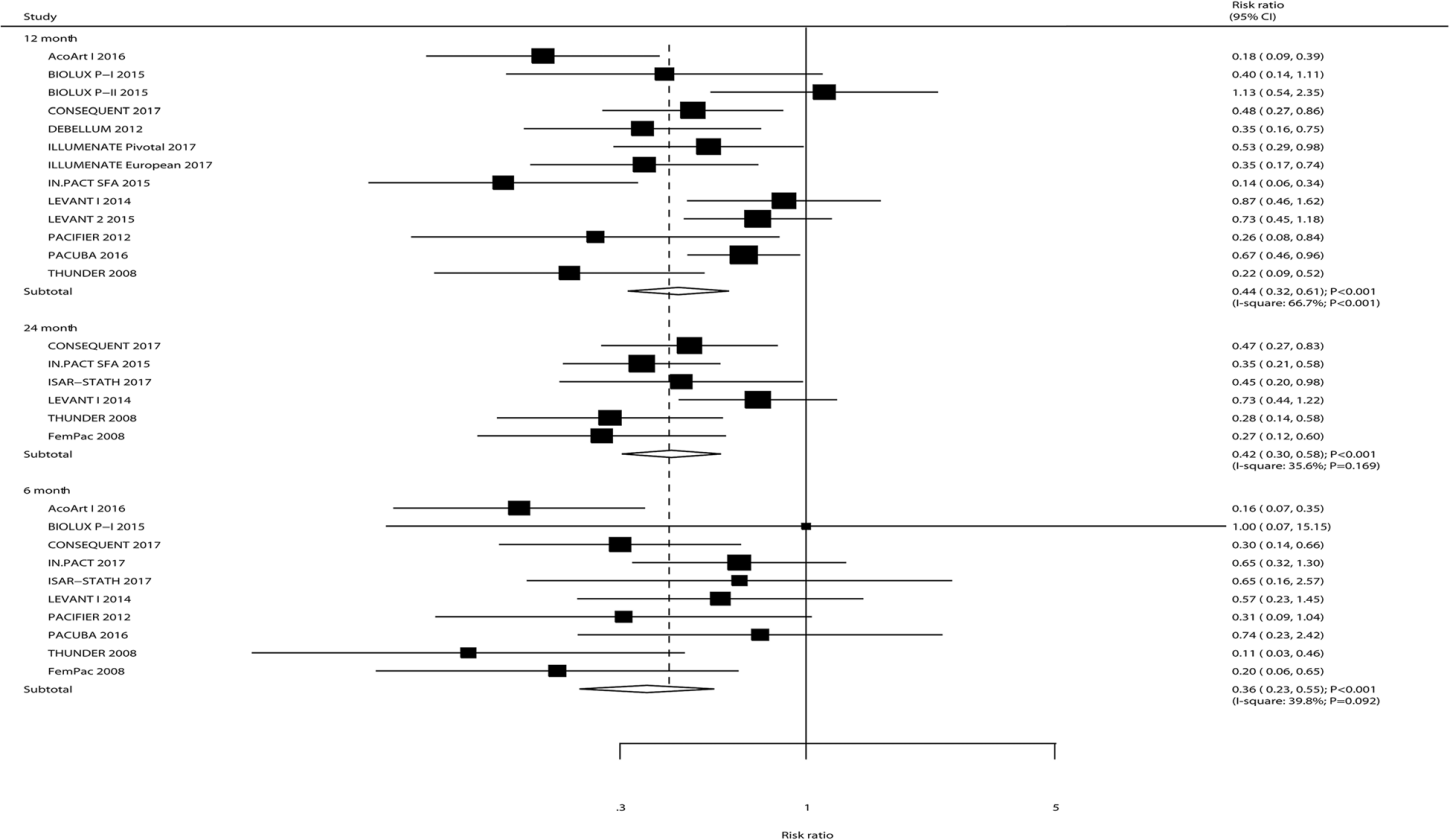
DEB versus UCB on the risk of TLR

The number of trials that reported TLR after 6, 12, and 24 months was 10, 13, and 6 trials, respectively. The pooled results indicated that the risk of TLR after 6 months (RR: 0.36; 95%CI: 0.23–0.55; *P* < 0.001), 12 months (RR: 0.44; 95%CI: 0.32–0.61; *P* < 0.001), and 24 months (RR: 0.42; 95%CI: 0.30–0.58; *P* < 0.001) are significantly reduced in patients who received DEB (Fig. 6). There was significant heterogeneity among the included trials for TLR after 6 and 12 months. Sensitivity analyses indicated that DEB versus UCB on the risk of TLR after 6 months, 12 months, and 24 months are unchanged after the sequential exclusion of any individual trial (Supplemental 1). The results of meta-regression indicated smoker percent (*P* = 0.035) hyperlipidemia percent (*P* = 0.041), and paclitaxel dose (*P* = 0.039) could affect the treatment on TLR after 6 months (Table 2). These significant differences between DEB and UCB on TLR risk are observed in most of the subsets. However, DEB did not yield any additional beneficial information on TLR after 6 months if the mean age of patients was ≥70.0 years, DM percent was ≥50.0%, and treated with 2.0 μg/mm^2^of paclitaxel. Moreover, the risk of TLR after 12 months between DEB and UCB showed no statistical significance, irrespective of the mean age of patients, and DM percent ≥50.0%. There were no significant publication biases for TLR after 6 months (*P*-value for Egger: 0.994; *P* value for Begg: 0.721) and 24 months (*P*-value for Egger: 0.269; *P* value for Begg: 0.260), whereas potential publication bias might exist for TLR after 12 months (*P*-value for Egger: 0.024; *P* value for Begg: 0.044; Supplemental 2).

### All-cause mortality

**Fig. 7:**
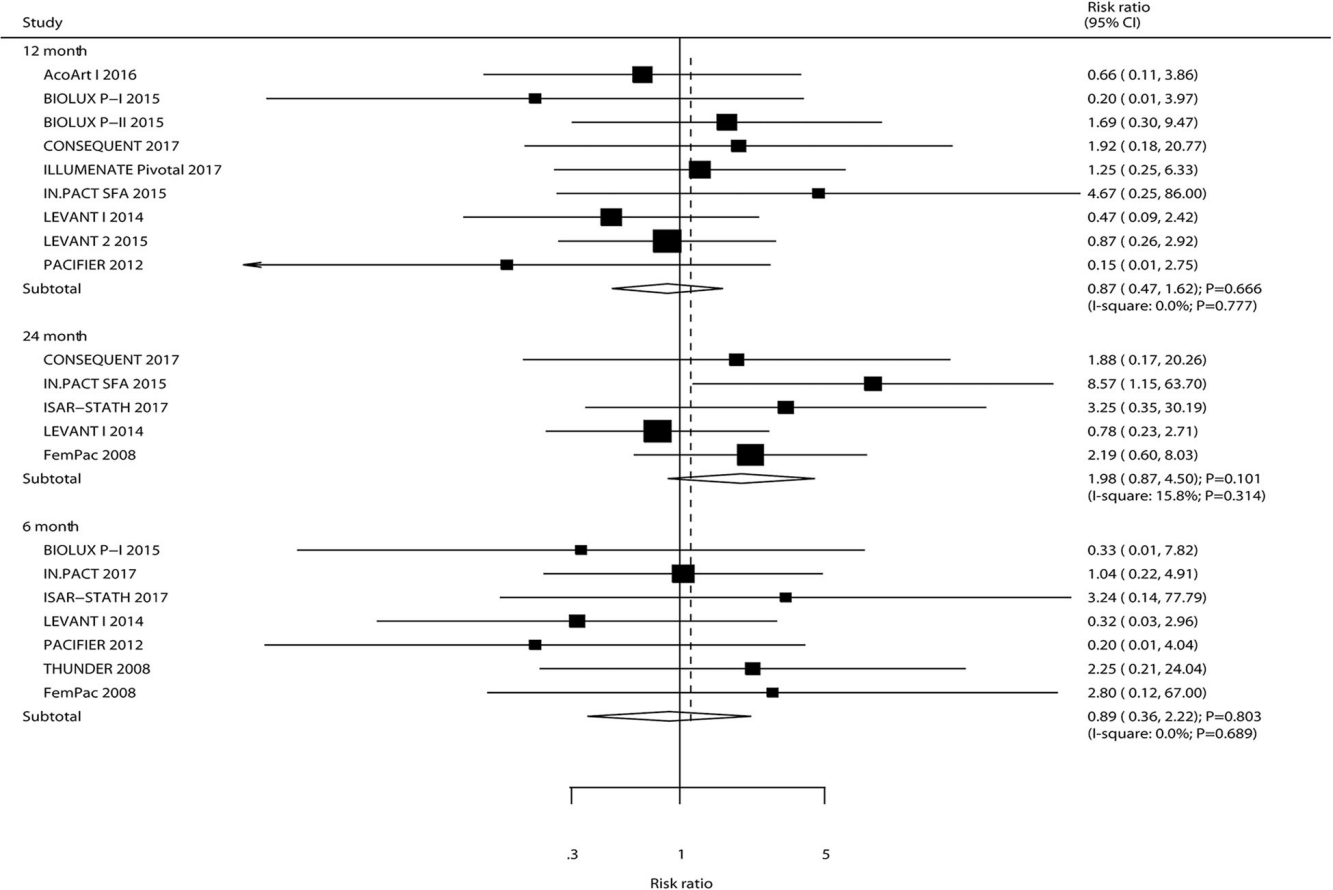
DEB versus UCB on the risk of all-cause mortality

The number of trials that reported all-cause mortality after 6, 12, and 24 months was seven, nine, and five, respectively. There were no significant differences between DEB and UCB for the risk of all-cause mortality after 6 months (RR: 0.89; 95%CI: 0.36–2.22; *P* = 0.803), 12 months (RR: 0.87; 95%CI: 0.47–1.62; *P* = 0.666), and 24 months (RR: 1.98; 95%CI: 0.87–4.50; *P* = 0.101) (Fig. 7). No significant heterogeneity among the included trials was observed. The results of sensitivity analyses indicated no significant differences between DEB and UCB on the risk of all-cause mortality after 6 months, 12 months, and 24 months (Supplemental 1). The pre-defined factors could not affect all-cause mortality after 6 months and 12 months by using meta-regression analyses (Table 2). No significant differences between DEB and UCB were observed on the risk of all-cause mortality after 6 and 12 months based on pre-defined factors. No significant publication bias for all-cause mortality after 6 months (*P*-value for Egger: 0.976; *P* value for Begg: 0.368) and 12 months (*P*-value for Egger: 0.748; *P* value for Begg: 0.754) were detected (Supplemental 2).

### Major adverse events

**Fig. 8:**
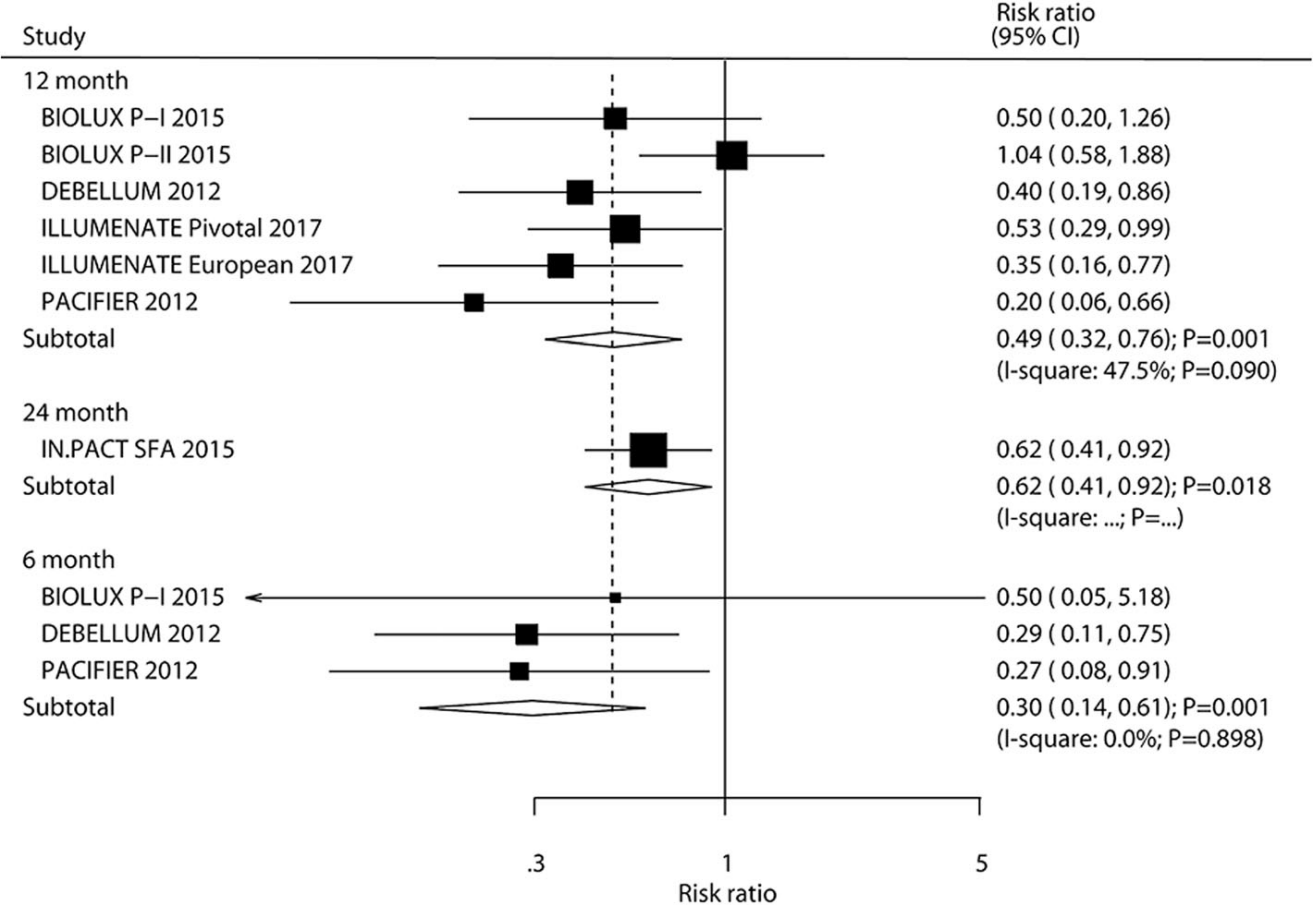
DEB versus UCB on the risk of major adverse events

The number of trials that reported major adverse events after 6, 12, and 24 months was three, six, and one, respectively. The results revealed that DEB significantly reduced the risk of major adverse events after 6 months (RR: 0.30; 95%CI: 0.14–0.61; *P* = 0.001), 12 months (RR: 0.49; 95%CI: 0.32–0.76; *P* = 0.001), and 24 months (RR: 0.62; 95%CI: 0.41–0.92; *P* = 0.018), and significant heterogeneity was observed for major adverse events after 12 months (Fig. 8). The risk of major adverse events after 12 months was unchanged after excluding individual trials (Supplemental 1). DM percent (*P* = 0.018), hypertension percent (*P* = 0.018), and paclitaxel dose (*P* = 0.046) could bias the affect of treatment of DEB on major adverse events after 12 months (Table 2). Although significant differences between DEB and UCB on major adverse events after 12 months were observed in most of the study subsets, DEB showed no significant effect on major adverse events after 12 months when the mean age of patients was ≥70.0 years, DM percent was ≥50.0%, hypertension percent was ≥80.0%, and treated with 3.0 μg/mm^2^ of paclitaxel. No significant publication bias for major adverse events after 12 months was observed (*P*-value for Egger: 0.064; *P* value for Begg: 0.060; Supplemental 2).

### Target lesion thrombosis

**Fig. 9:**
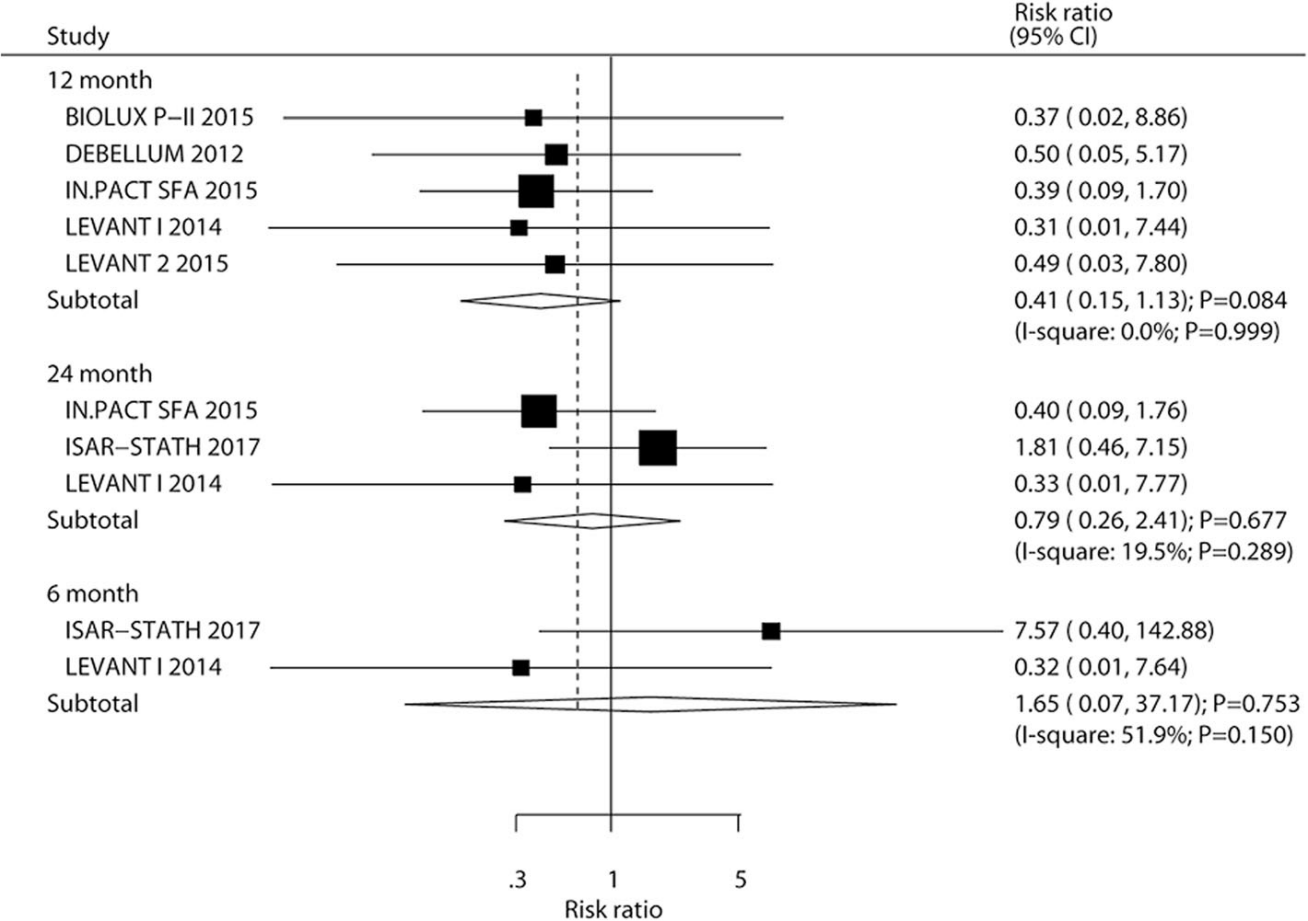
DEB versus UCB on the risk of target lesion thrombosis

The number of trials that reported target lesion thrombosis after 6, 12, and 24 months was two, five, and three, respectively. The summary RRs indicated that DEB did not yield any additional benefits on target lesion thrombosis after 6 months (RR: 1.65; 95%CI: 0.07–37.17; *P* = 0.753), 12 months (RR: 0.41; 95%CI: 0.15–1.13; *P* = 0.084), and 24 months (RR: 0.79; 95%CI: 0.26–2.41; *P* = 0.677), and no significant heterogeneity was observed among the included trials (Fig. 9). Sensitivity, subgroup, and publication biases were not conducted due to smaller number of included trials.

### Amputation

**Fig. 10:**
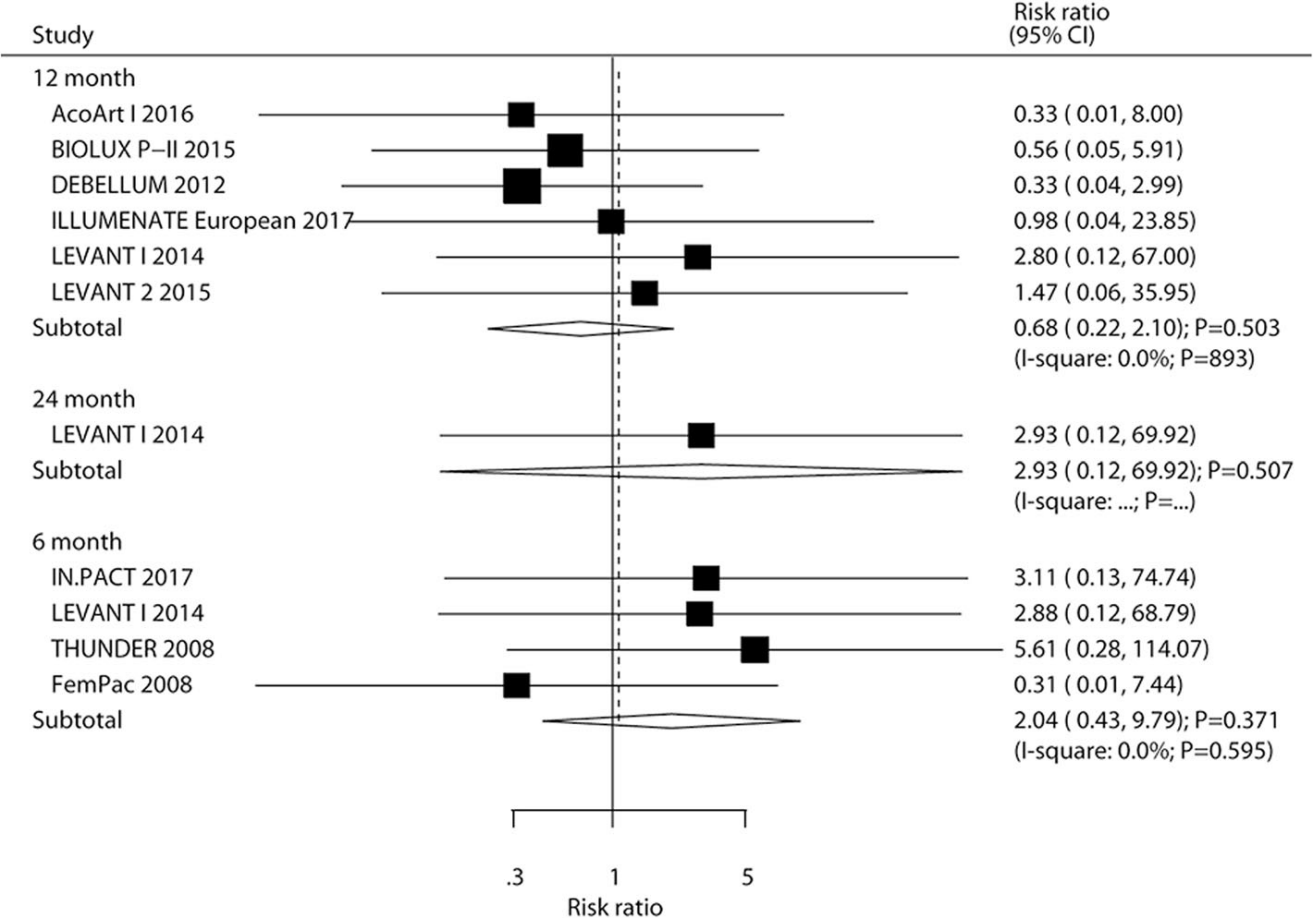
DEB versus UCB on the risk of amputation

The number of trials that reported amputation after 6, 12, and 24 months was four, six, and one, respectively. There were no significant differences between DEB and UCB groups regarding the risk of amputation after 6 months (RR: 2.04; 95%CI: 0.43–9.79; *P* = 0.371), 12 months (RR: 0.68; 95%CI: 0.22–2.10; *P* = 503), and 24 months (RR: 2.93; 95%CI: 0.12–69.92; *P* = 0.507). There was no evidence of heterogeneity among the included trials (Fig. 10). Sensitivity analysis indicated that the risk of amputation after 12 months was stable and unchanged after sequential exclusion of any individual trial (Supplemental 1). The results of subgroup analyses for amputation after 12 months remained consistent with the overall analysis (Table 2). No significant publication bias for amputation after 12 months was observed (*P*-value for Egger: 0.167; *P* value for Begg: 0.260; Supplemental 2).

## Discussion

This systematic review and meta-analysis based on RCTs explored potential treatment effects of DEB versus UCB in patients with femoropopliteal arterial occlusive disease. This quantitative study included 2706 patients from 17 RCTs with varied patient characteristics. The results of this meta-analysis indicated that DEB was superior to UCB and showed significant improvements in MLD, LLL, primary patency, restenosis, TLR, and major adverse events. However, patients who received DEB showed no beneficial effects on all-cause mortality, target lesion thrombosis, and amputation than those who received UCB. Moreover, the treatment effects of DEB on MLD at 6 months might differ by mean age, smoker percent, and paclitaxel dose, while the effects on LLL differed based on mean age, smoker percent, DM percent, hyperlipidemia percent, hypertension, and paclitaxel dose. Furthermore, at 6 months, mean age could affect the treatment on restenosis; and smoker percent and paclitaxel dose could bias the effects on restenosis and TLR. At 12 months, the risk of primary patency, and major adverse events could affect by DM percent and paclitaxel dose. Hyperlipidemia percent could bias the treatment effects on primary patency at 12 months, restenosis at 6 months, and TLR at 6 months. The risk of restenosis at 6 months and major adverse events at 12 months differed based on hypertension percent.

Several systematic reviews and meta-analyses have already addressed the treatment effects of DEB versus UCB in patients with femoropopliteal arterial occlusive disease. Cassese et al. have conducted a meta-analysis of 4 RCTs, and the results suggested that paclitaxel-coated balloon significantly reduced the risk of TLR, angiographic restenosis, and LLL, whereas no significant difference was observed between the paclitaxel-coated balloon and UCB for the risk of mortality [34]. Several other important indexes were not reported, and whether the treatment effects differed based on the characteristics of patients were not evaluated. The study conducted by Kayssi et al. have indicated that DEB versus UCB showed an advantage on primary patency, restenosis rate, and TLR after follow-up for 12 months in patients with PAD in the lower limbs, whereas no significant differences were observed between DEB and UCB for the risk of amputation, all-cause mortality, and change in ABI and Rutherford category after follow-up for 12 months [35]. However, the investigated outcomes after 6 and 24 months were not reported. Giacoppo et al. have conducted an updated meta-analysis of 8 RCTs and pointed out that DEB significantly reduced the risk of TLR, while showed no significant effect on all-cause mortality, irrespective of mid-term or long-term follow-up duration [36]. However, several other endpoints and results based on patient characteristics were not evaluated. Jaff et al. have conducted a network meta-analysis to compare the optimal endovascular strategy in patients with femoropopliteal arterial occlusive disease, and the results showed that DEB improved the TLR rates as compared with percutaneous transluminal angioplasty and bare-metal stent [37]. However, the study did not illustrate other clinical endpoints. Katsanos et al. have summarized 28 RCTs with 4663 patients who received paclitaxel-coated/paclitaxel-eluting stent or balloon vs. controls for femoropopliteal arterial occlusive disease and showed that the risk of death at 2 and 5 years was higher in patients who received a paclitaxel-coated/paclitaxel-eluting stent or balloon when compared with controls [38]. In addition, their meta-regression analysis demonstrated that exposure to paclitaxel showed association with absolute risk of death. Therefore, the selection of DEB seems crucial in determining the benefits of DEB, and future studies should consider this point. A meta-analysis conducted by Klumb et al. have shown association of DEB with increased incidence of freedom from TLR at 12 and 24 months. Moreover, DEB significantly increased the risk of 2-year mortality [39]. However, whether the treatment effects between DEB and UCB differed according to the patients’ characteristics were not illustrated. Therefore, the current systematic review and meta-analysis was conducted to update the efficacy and safety of DEB versus UCB for patients with femoropopliteal arterial occlusive disease.

The pooled results indicated that DEB significantly increased the MLD level, and the heterogeneity among the included trials might be due to BIOLUX P-II and CONSEQUENT trials. Moreover, the pooled results of LLL are affected by BIOLUX P-II and PACIFIER trials. The characteristics of patients, including older age, a higher percent of smokers, DM, hyperlipidemia, and hypertension, were associated with poor prognosis of patients with femoropopliteal arterial occlusive disease [2, 40–42]. Moreover, the mean length of lesions and paclitaxel dose might affect the treatment effects of DEB and UCB in patients with femoropopliteal arterial occlusive disease. In addition, the sample size among the included studies could affect the weighted mean from the pooled results, and the stability of results from individual trials was relatively highly variable. Furthermore, the number of studies included in the subgroups was imbalanced, explaining significant difference between DEB and UCB for LLL in those with hyperlipidemia percent of ≥60.0%. Finally, significant improvement in MLD and LLL might be due to paclitaxel inhibition of proliferation of smooth muscle cells.

We noted that DEB versus UCB yielded additional beneficial effects on primary patency, restenosis, TLR, and major adverse events, whereas no significant effect was observed on all-cause mortality, target lesion thrombosis, and amputation. Patients who received local paclitaxel have the ability to inhibit neointimal proliferation, and this biological effect showed association with significant improvements in Rutherford class and ABI, leading to a lower risk of TLR. Moreover, the treatment effects of DEB are more outstanding in younger patients, and a low percentage of smokers, DM, hyperlipidemia, hypertension, and high paclitaxel dose. The potential reason for this could be that the prognosis of femoropopliteal arterial occlusive disease in elderly remained poor. Moreover, smoking increased the risk of asymptomatic PAD, and the prognosis of femoropopliteal arterial occlusive disease might be affected by accumulative amount of smoking [41]. In addition, hyperlipidemia and hypertension are significant risk factors for atherosclerosis, affecting the prognosis of femoropopliteal arterial occlusive disease. Although no significant difference between DEB and UCB for target lesion thrombosis, and amputation was found, these results still needed further verification due to the occurrence of smaller events and hence a long-term follow-up of the study is warranted. Finally, no significant difference between DEB and UCB for the risk of all-cause mortality was detected, which was inconsistent with prior meta-analyses [38, 39]. An important individual patient data meta-analysis also found that paclitaxel-containing devices yielded an absolute of 4.6% increased mortality risk for patients with patients with symptomatic femoropopliteal peripheral artery disease [43]. However, a real-world safety analysis found that paclitaxel-based drug-eluting devices showed no association with the risk of all-cause mortality for over 11 years [44]. In our study, all included studies applied paclitaxel coated balloon, and late paclitaxel toxicity could explain the increased risk of all-cause mortality after 12 months of prior studies [38, 39, 43]. Several reasons could be explained due to no significant difference between groups for the risk of all-cause mortality in our study: (1) the study conducted by Katsanos et al. have used paclitaxel-coated/paclitaxel-eluting stent or balloon as intervention, and the result was more robust than DEB alone [38]; (2) the analyses of prior meta-analysis applied fixed-effects model, and the results did not consider the variations across the included trials, and the results are more radical [38, 39]; and (3) the individual patient data meta-analysis with 4-year median follow-up, and expected events occurred were enough to detect minor differences between the groups.

There were several strengths in this study that should be highlighted: (1) the current study is based on RCTs, which avoided overestimation of treatment effects of DEB versus UCB concerns of observational studies; (2) the comprehensive results regarding DEB versus UCB were reported based on the follow-up duration; (3) stratified analyses according to the patients’ characteristics were conducted to evaluate the treatment effects of DEB versus UCB in specific subpopulations; and (4) *P* values between subgroups were calculated to compare the treatment effects according to the patients’ characteristics.

However, our study has some limitations that should be acknowledged. Firstly, various types of interventions and controls might affect the net treatment effects between DEB and UCB. Secondly, stratified analyses, according to the length of the lesion were not calculated as several studies did not report the length of the lesion. Thirdly, the current study based on published articles and unpublished data were unavailable. Finally, this study used pooled data extracted from individual trials, restricting the detailed analyses according to the patients’ characteristics.

In conclusion, the current study suggested that DEB was superior over UCB for patients with femoropopliteal arterial occlusive disease in terms of MLD, LLL, primary patency, restenosis, TLR, and major adverse events, whereas the risk of all-cause mortality, target lesion thrombosis, and amputation required further long-term follow-up RCTs to verify the treatment effects between DEB and UCB.

## Supplementary information

**Additional file 1: Figure S1.** Sensitivity analysis for MLD at 6 months. **Figure S2.** Sensitivity analysis for LLL at 6 months. **Figure S3.** Sensitivity analysis for primary patency at 12 months. **Figure S4.** Sensitivity analysis for restenosis at 6 months. **Figure S5.** Sensitivity analysis for TLR at 6 months. **Figure S6.** Sensitivity analysis for TLR at 12 months. **Figure S7.** Sensitivity analysis for TLR at 24 months. **Figure S8.** Sensitivity analysis for all-cause mortality at 6 months. **Figure S9.** Sensitivity analysis for all-cause mortality at 12 months. **Figure S10.** Sensitivity analysis for major adverse events at 12 months. **Figure S11.** Sensitivity analysis for amputation at 12 months.

**Additional file 2: Figure S12.** Funnel plot for MLD at 6 months. **Figure S13.** Funnel plot for LLL at 6 months. **Figure S14.** Funnel plot for primary patency at 12 months. **Figure S15.** Funnel plot for restenosis at 6 months. **Figure S16.** Funnel plot for TLR at 6 months. **Figure S17.** Funnel plot for TLR at 12 months. **Figure S18.** Funnel plot for TLR at 24 months. **Figure S19.** Funnel plot for all-cause mortality at 6 months. **Figure S20.** Funnel plot for all-cause mortality at 12 months. **Figure S21.** Funnel plot for major adverse events at 12 months. **Figure S22.** Funnel plot for amputation at 12 months.

## Data Availability

The datasets used and/or analysed during the current study are available from the corresponding author on reasonable request.

## Abbreviations

PAD: Peripheral artery disease
RCTs: Randomized controlled trials
DEB: Drug-eluting balloon
LLL: Late lumen loss
TLR: Target lesion revascularization
UCB: Uncoated balloon
PRISMA: Preferred reporting items for systematic reviews and meta-analysis
MLD: Minimal luminal diameter
DM: Diabetes mellitus
ABI: Ankle-brachial index
WMD: Weighted mean difference
CI: Confidence interval
RR: Relative risk
