# Supplementary material for: Comparison of efficacy and safety of drug-eluting versus uncoated balloon angioplasty for femoropopliteal arterial occlusive disease: a meta-analysis
Source: BMC Cardiovasc Disord. 2020 Aug 31;20:395. doi: 10.1186/s12872-020-01667-y (PMC7457510; doi:10.1186/s12872-020-01667-y)
Supplement: Supplementary file 1 — Additional file 1: Figure S1. Sensitivity analysis for MLD at 6 months. Figure S2. Sensitivity analysis for LLL at 6 months. Figure S3. Sensitivity analysis for primary patency at 12 months. Figure S4. Sensitivity analysis for restenosis at 6 months. Figure S5. Sensitivity analysis for TLR at 6 months. Figure S6. Sensitivity analysis for TLR at 12 months. Figure S7. Sensitivity analysis for TLR at 24 months. Figure S8. Sensitivity analysis for all-cause mortality at 6 months. Figure S9. Sensitivity analysis for all-cause mortality at 12 months. Figure S10. Sensitivity analysis for major adverse events at 12 months. Figure S11. Sensitivity analysis for amputation at 12 months. [file 12872_2020_1667_MOESM1_ESM.docx]

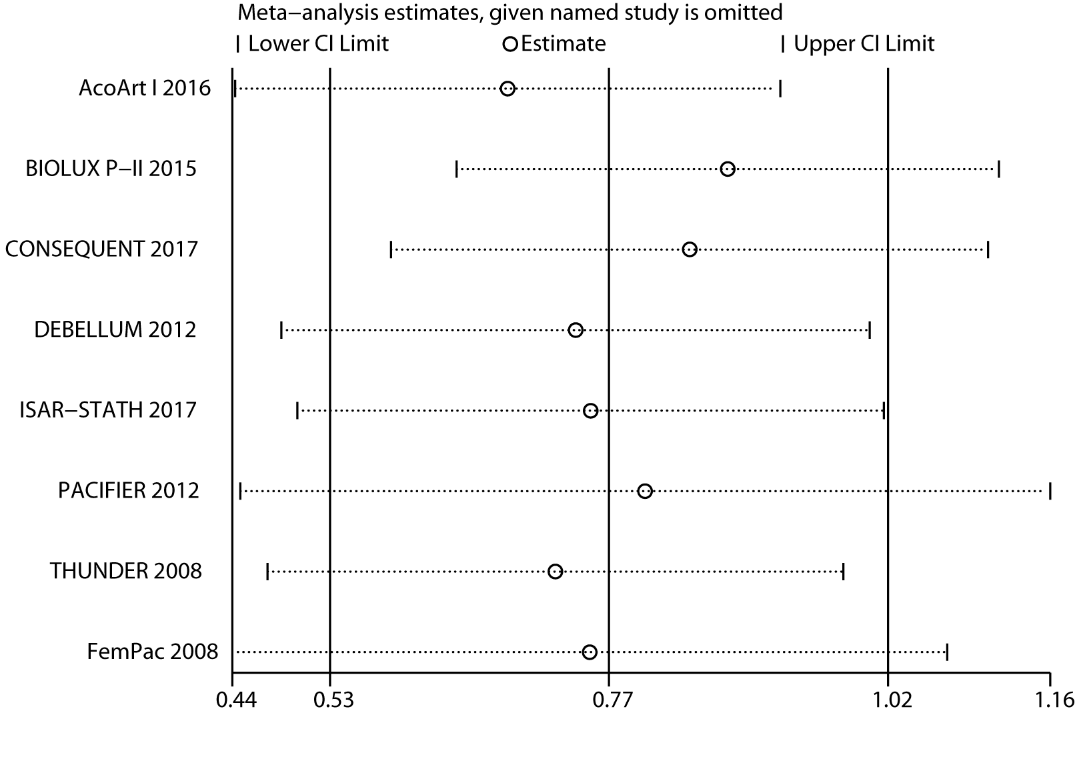


Figure S1. Sensitivity analysis for MLD at 6 months


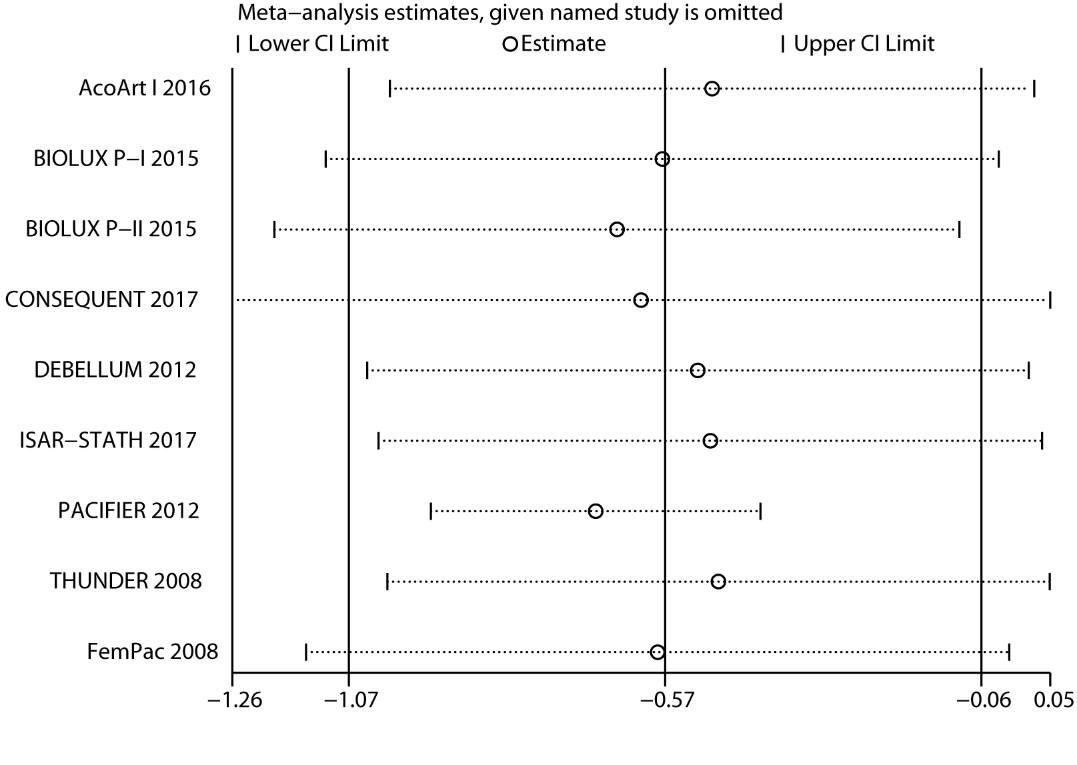


Figure S2. Sensitivity analysis for LLL at 6 months


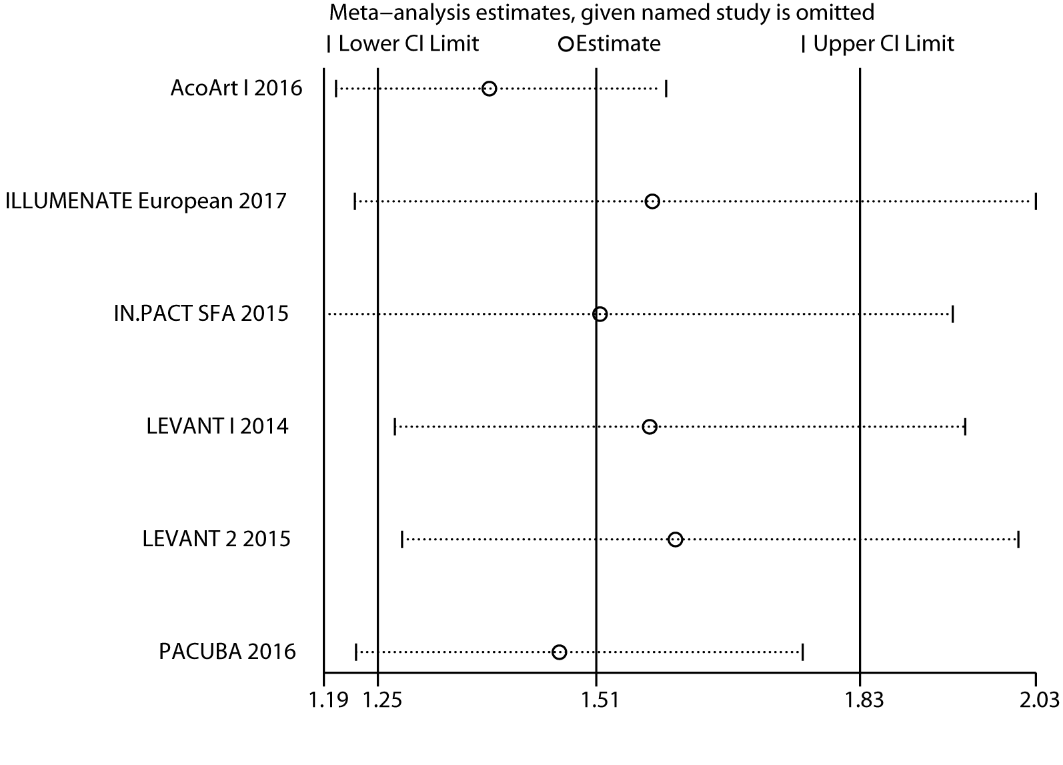


Figure S3. Sensitivity analysis for primary patency at 12 months


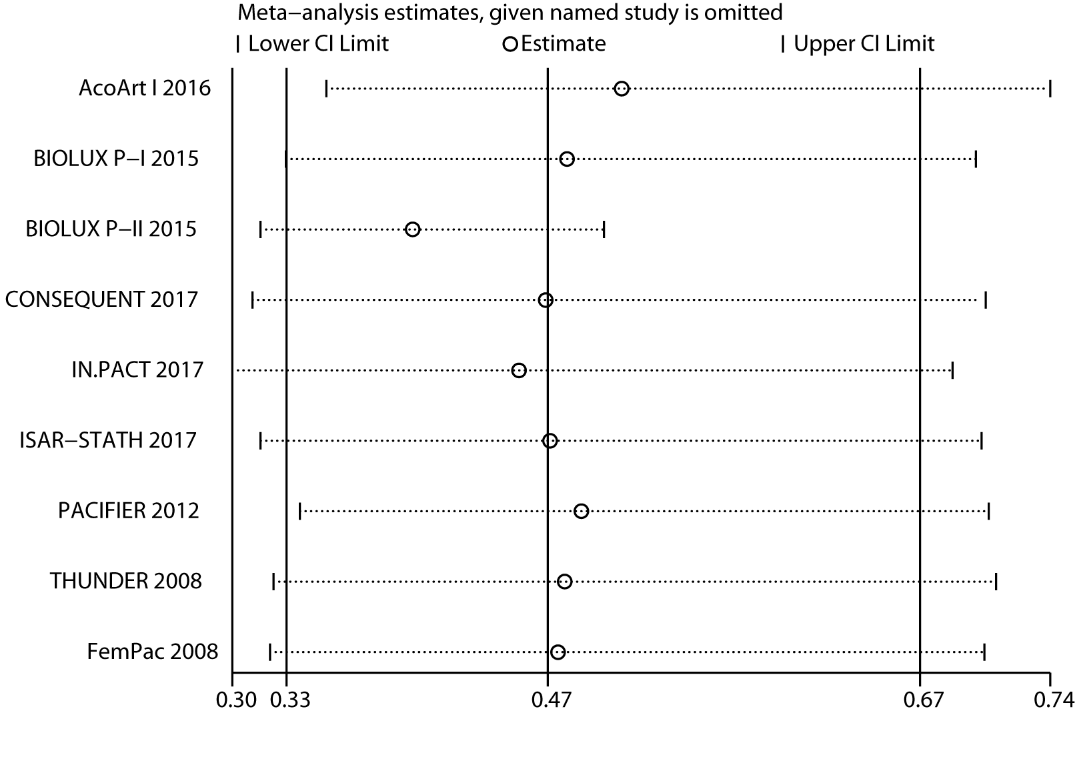


Figure S4. Sensitivity analysis for restenosis at 6 months


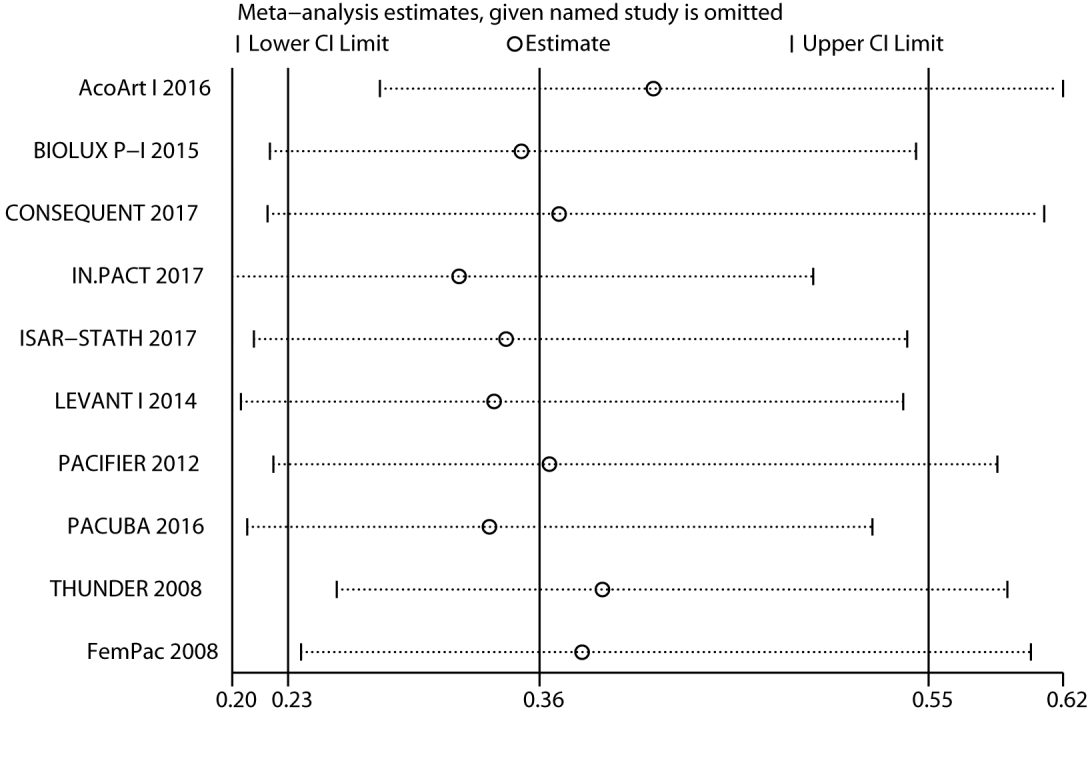


Figure S5. Sensitivity analysis for TLR at 6 months


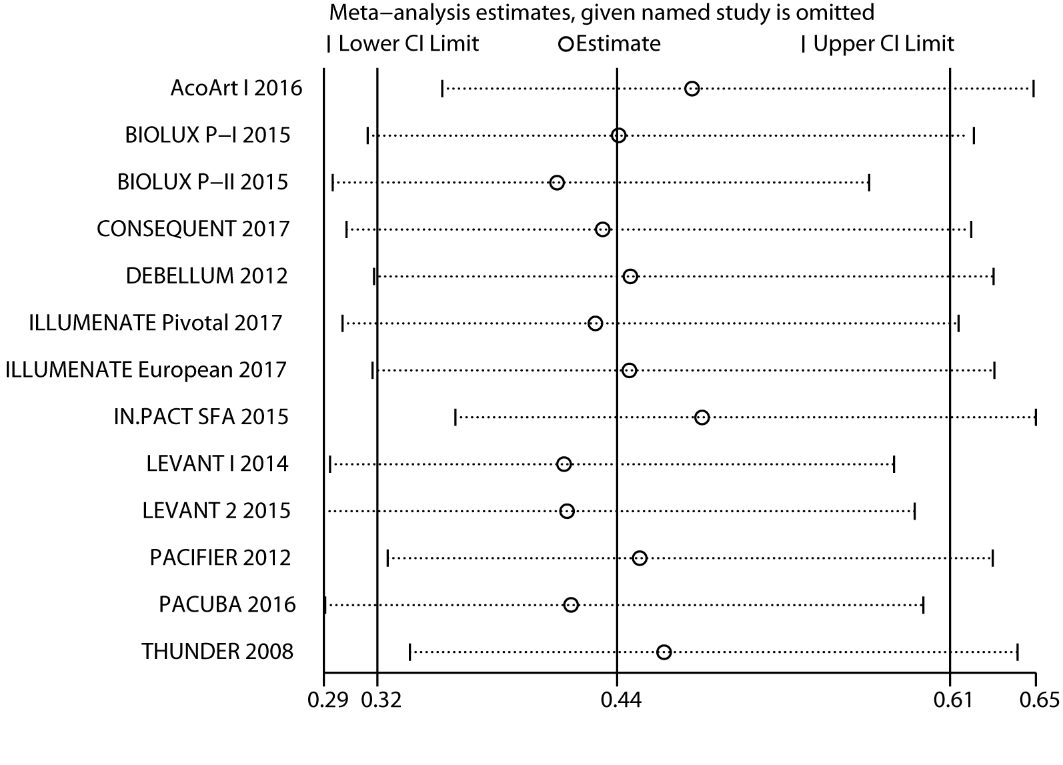


Figure S6. Sensitivity analysis for TLR at 12 months


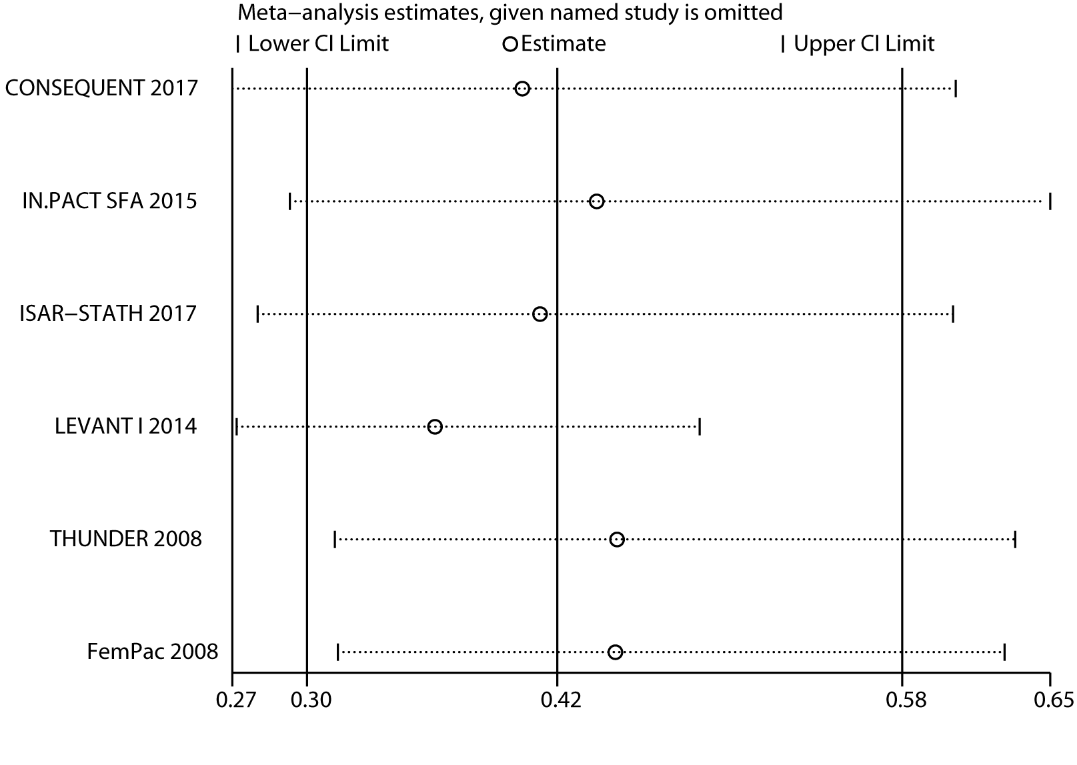


Figure S7. Sensitivity analysis for TLR at 24 months


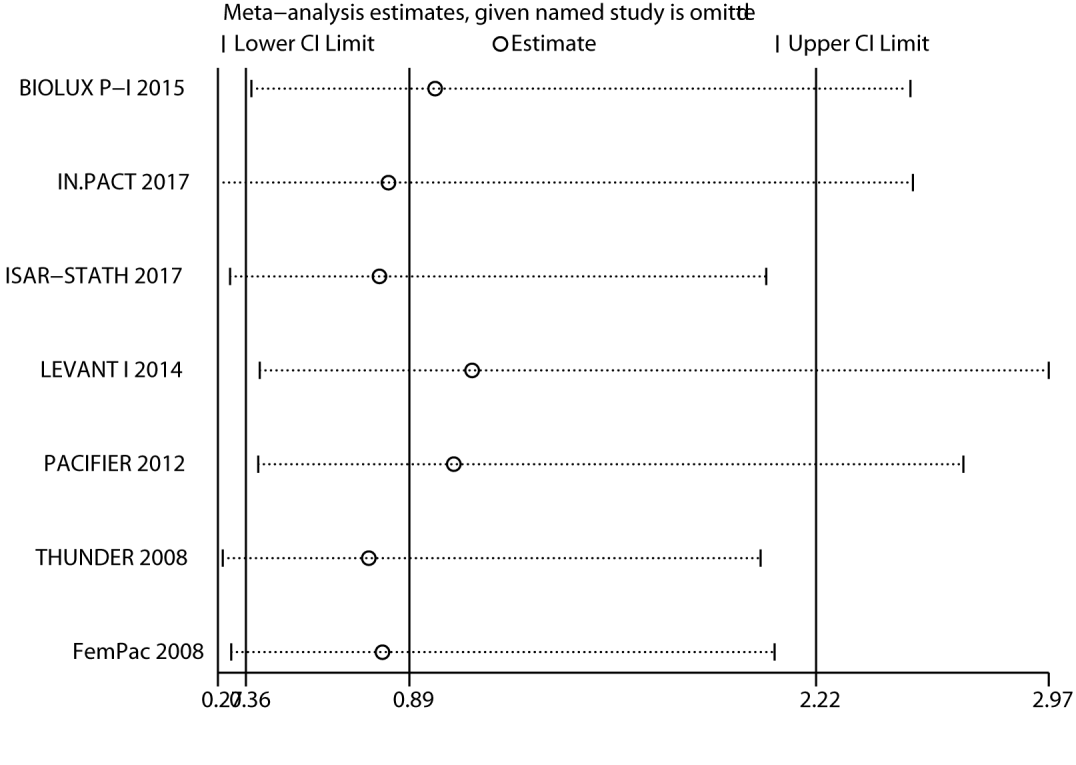


Figure S8. Sensitivity analysis for all-cause mortality at 6 months


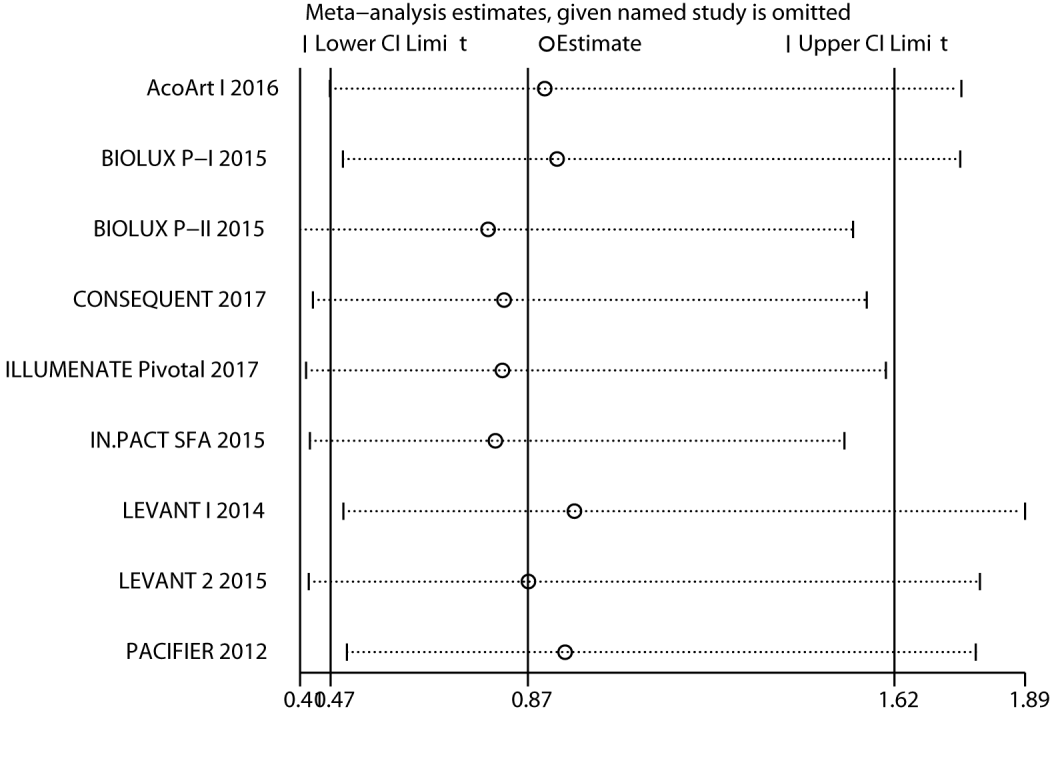


Figure S9. Sensitivity analysis for all-cause mortality at 12 months


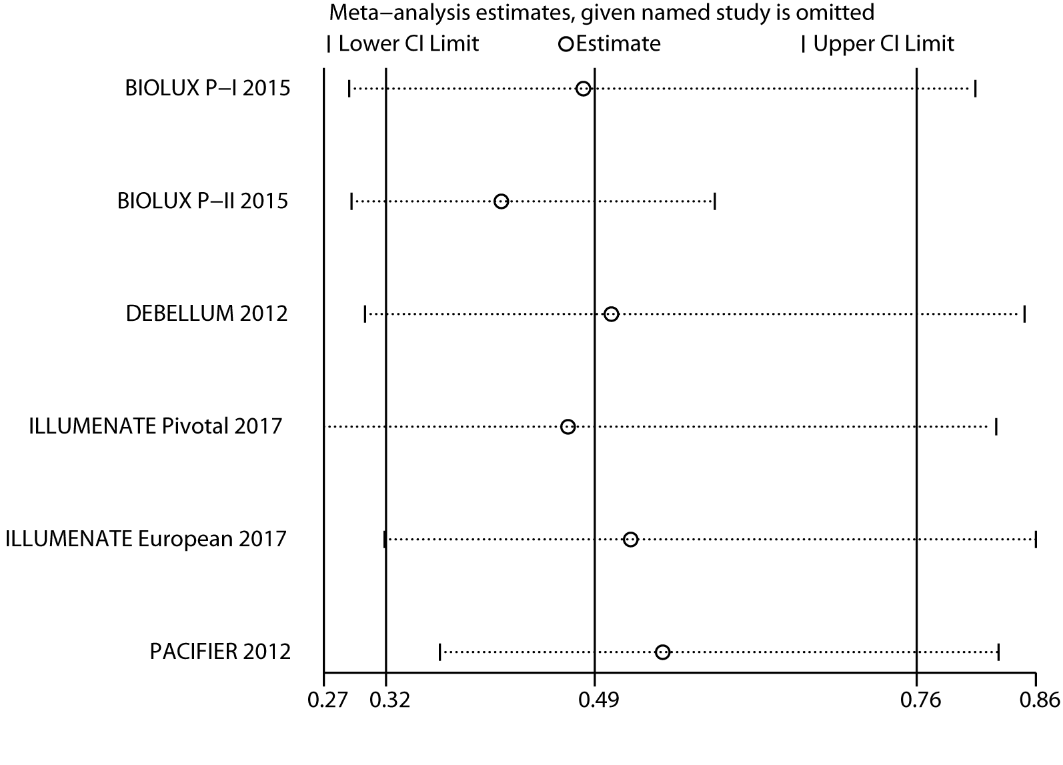


Figure S10. Sensitivity analysis for major adverse events at 12 months


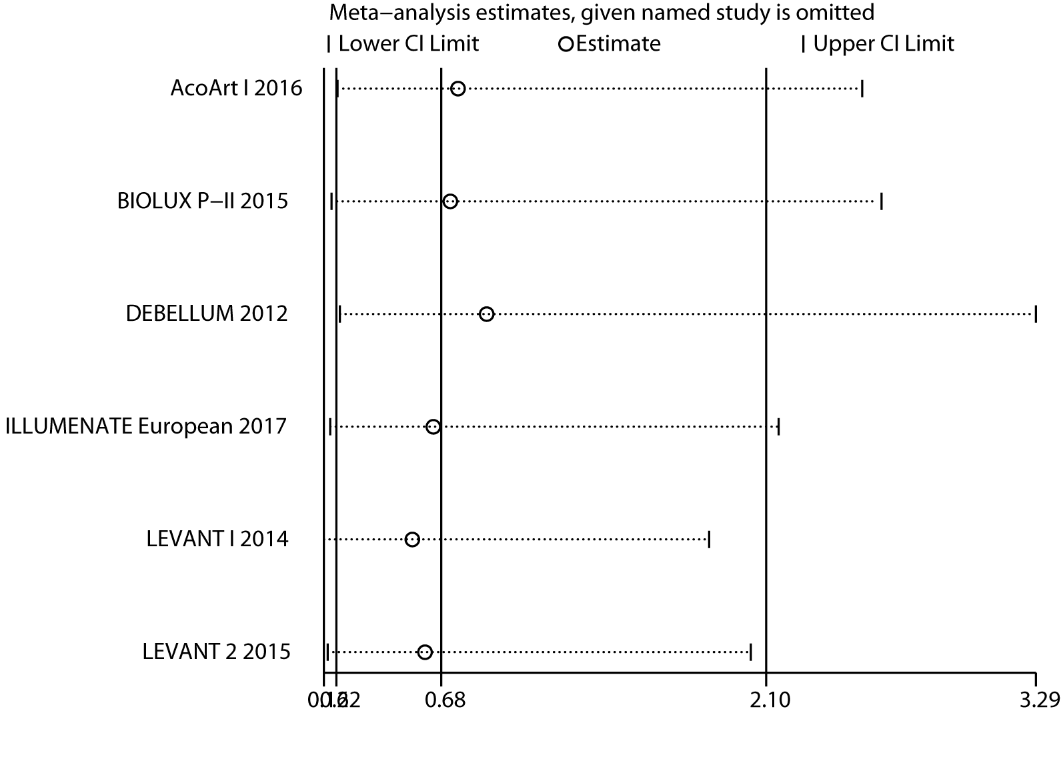


Figure S11. Sensitivity analysis for amputation at 12 months
