# Supplementary material for: Comparison of efficacy and safety of drug-eluting versus uncoated balloon angioplasty for femoropopliteal arterial occlusive disease: a meta-analysis
Source: BMC Cardiovasc Disord. 2020 Aug 31;20:395. doi: 10.1186/s12872-020-01667-y (PMC7457510; doi:10.1186/s12872-020-01667-y)
Supplement: Supplementary file 2 — Additional file 2: Figure S12. Funnel plot for MLD at 6 months. Figure S13. Funnel plot for LLL at 6 months. Figure S14. Funnel plot for primary patency at 12 months. Figure S15. Funnel plot for restenosis at 6 months. Figure S16. Funnel plot for TLR at 6 months. Figure S17. Funnel plot for TLR at 12 months. Figure S18. Funnel plot for TLR at 24 months. Figure S19. Funnel plot for all-cause mortality at 6 months. Figure S20. Funnel plot for all-cause mortality at 12 months. Figure S21. Funnel plot for major adverse events at 12 months. Figure S22. Funnel plot for amputation at 12 months. [file 12872_2020_1667_MOESM2_ESM.docx]

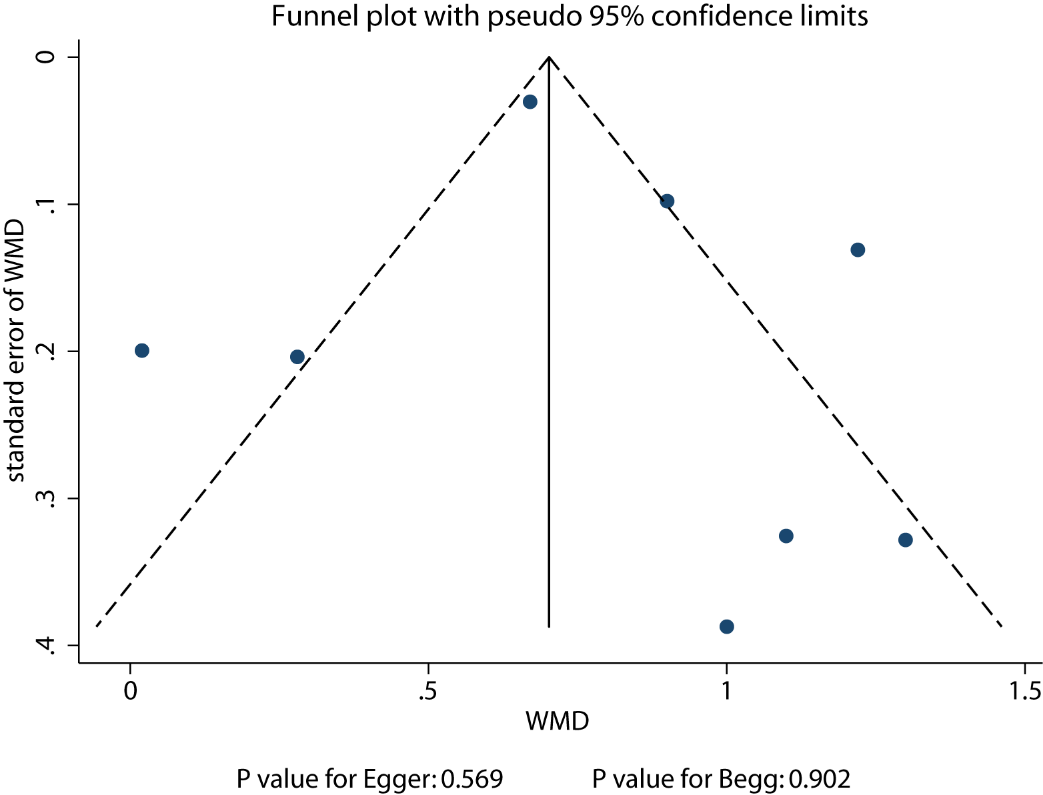


Figure S12. Funnel plot for MLD at 6 months


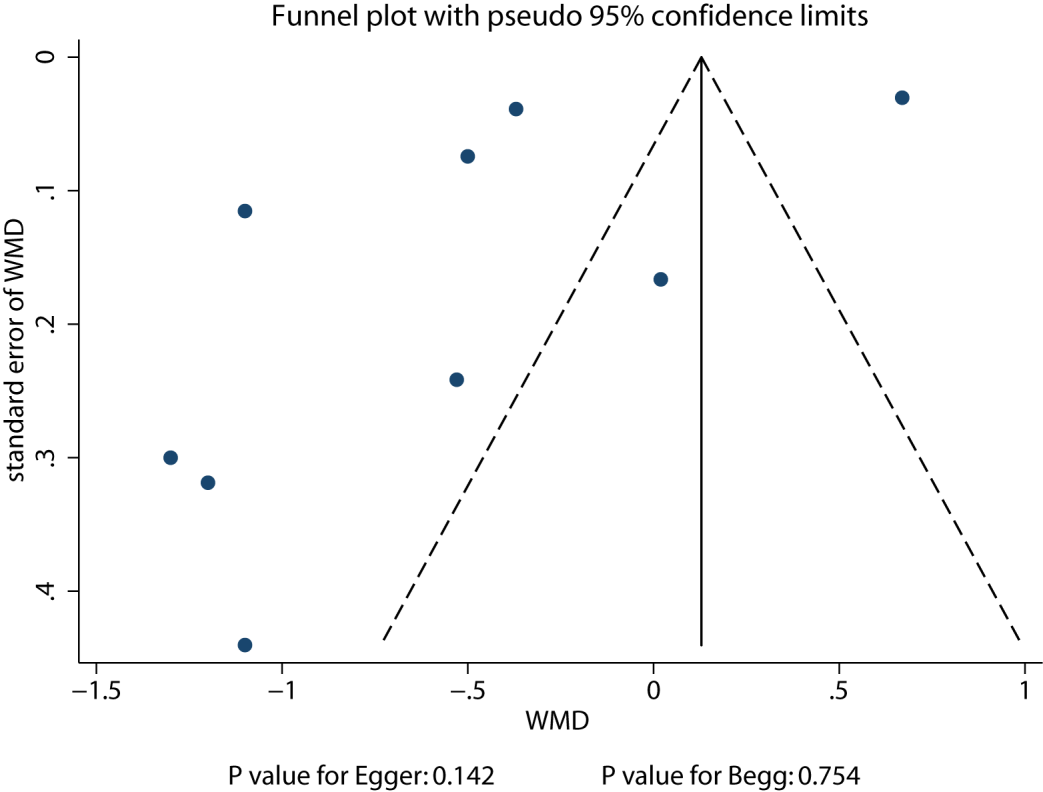


Figure S13. Funnel plot for LLL at 6 months


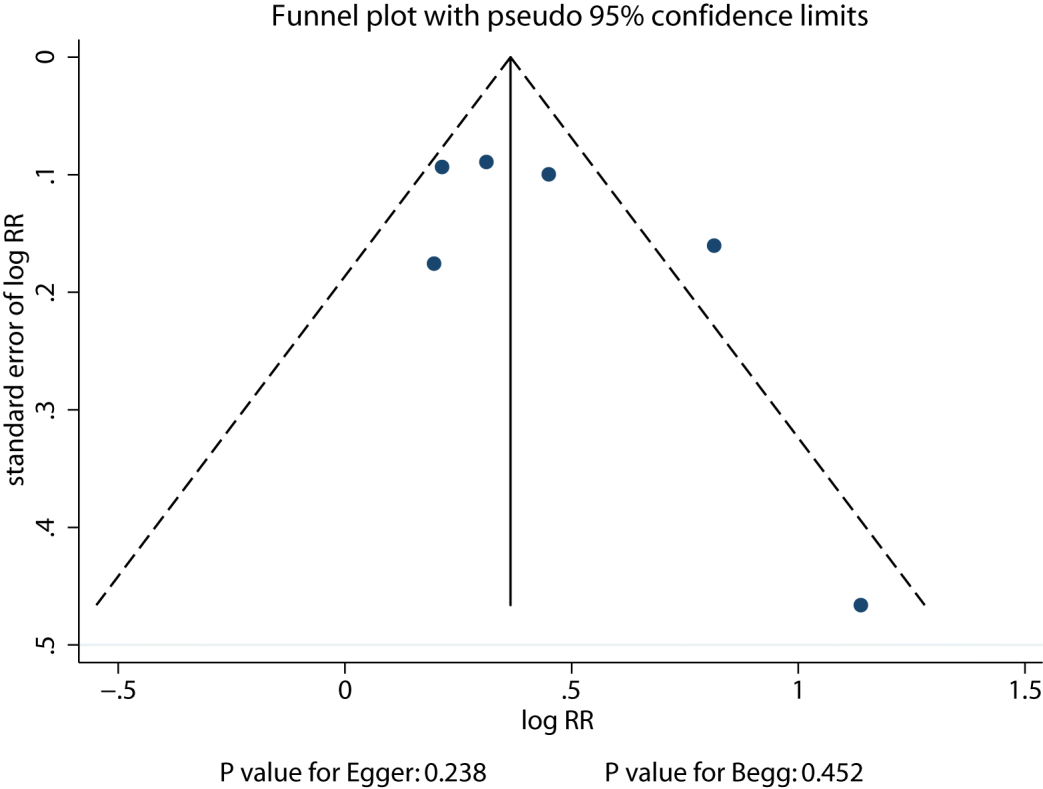


Figure S14. Funnel plot for primary patency at 12 months


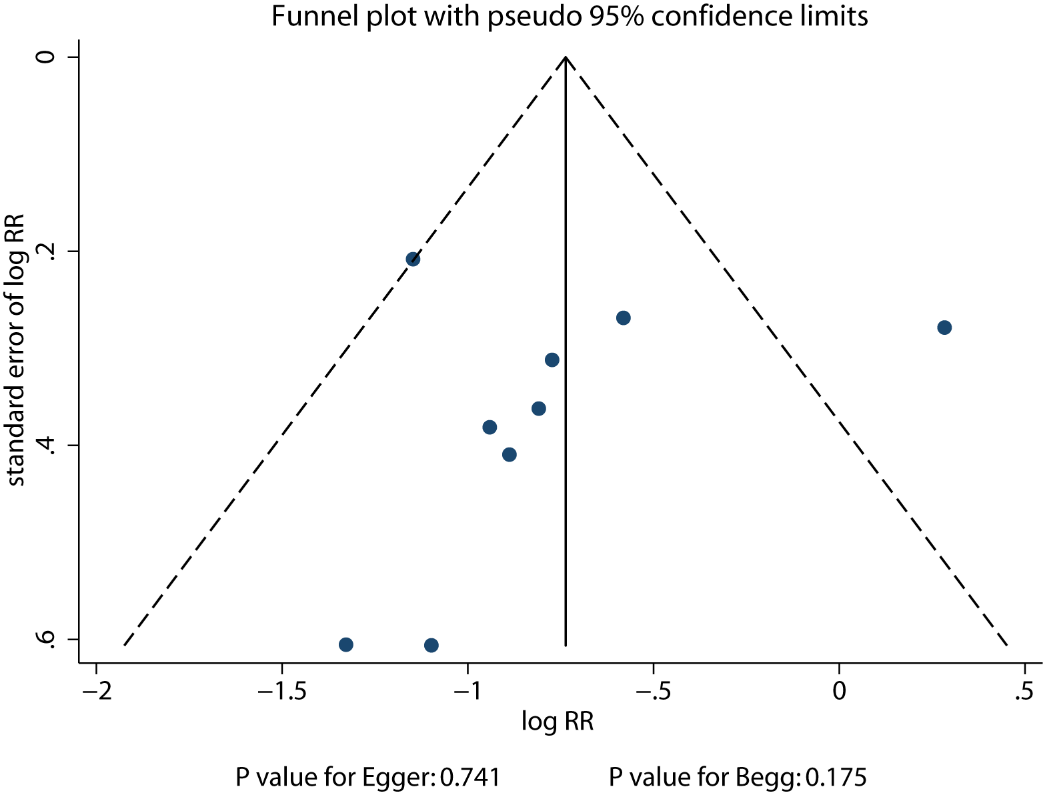


Figure S15. Funnel plot for restenosis at 6 months


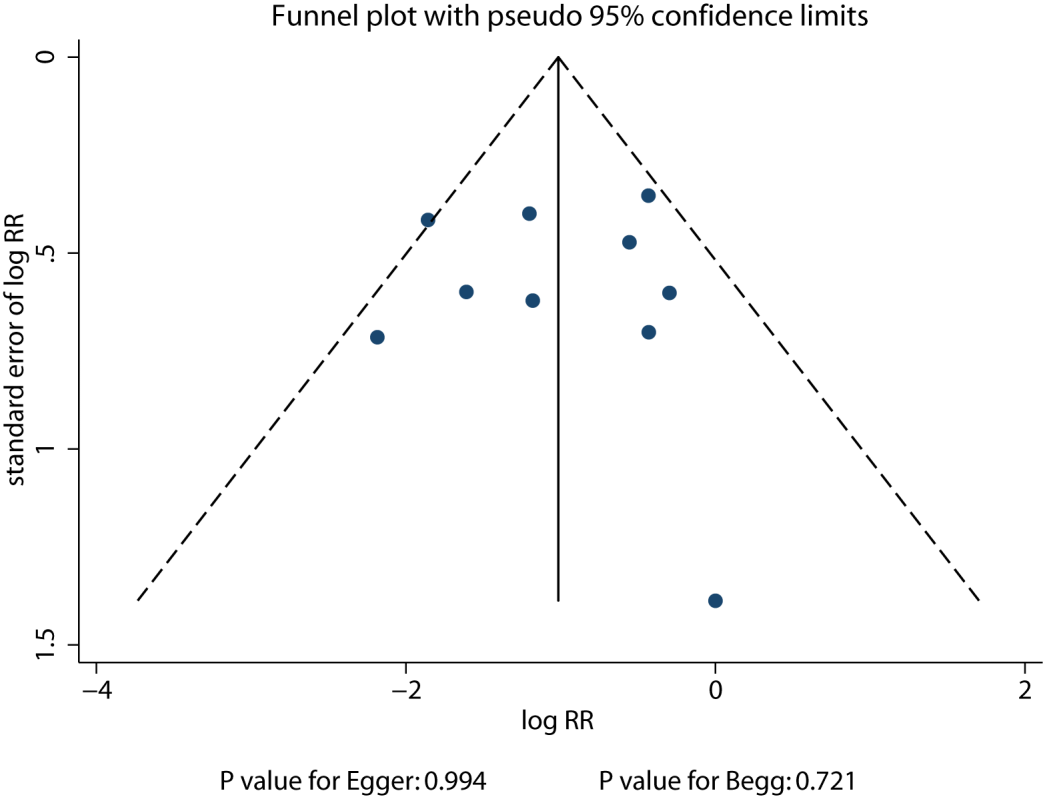


Figure S16. Funnel plot for TLR at 6 months


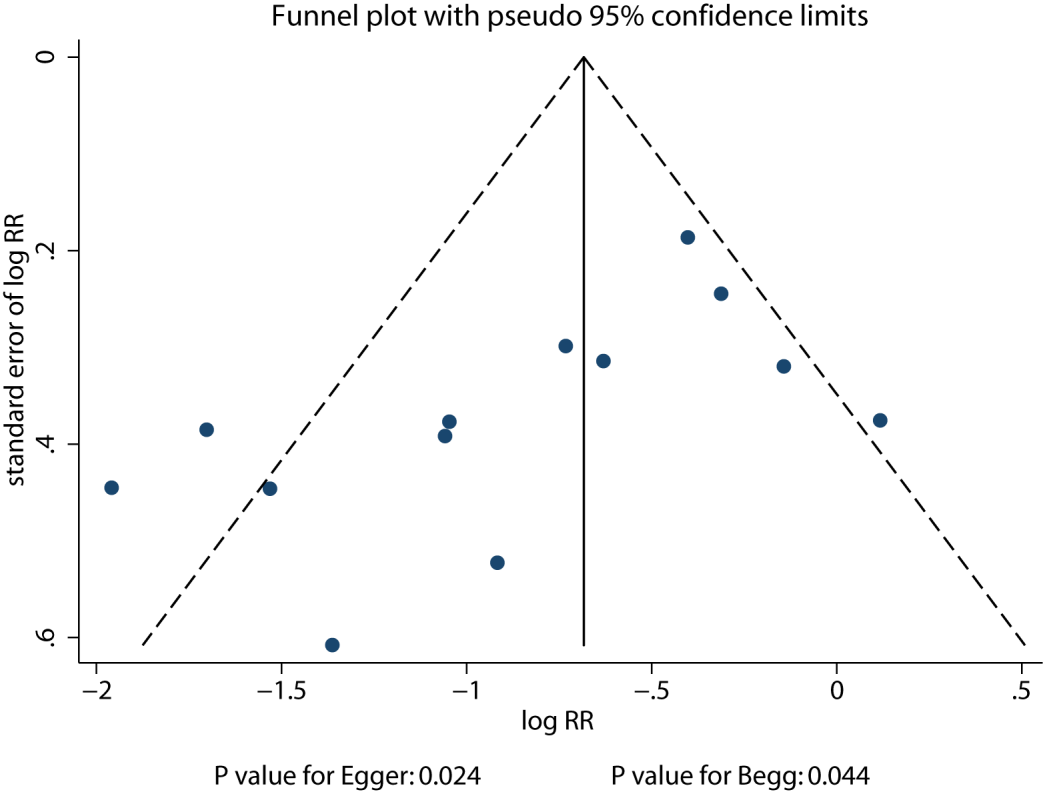


Figure S17. Funnel plot for TLR at 12 months


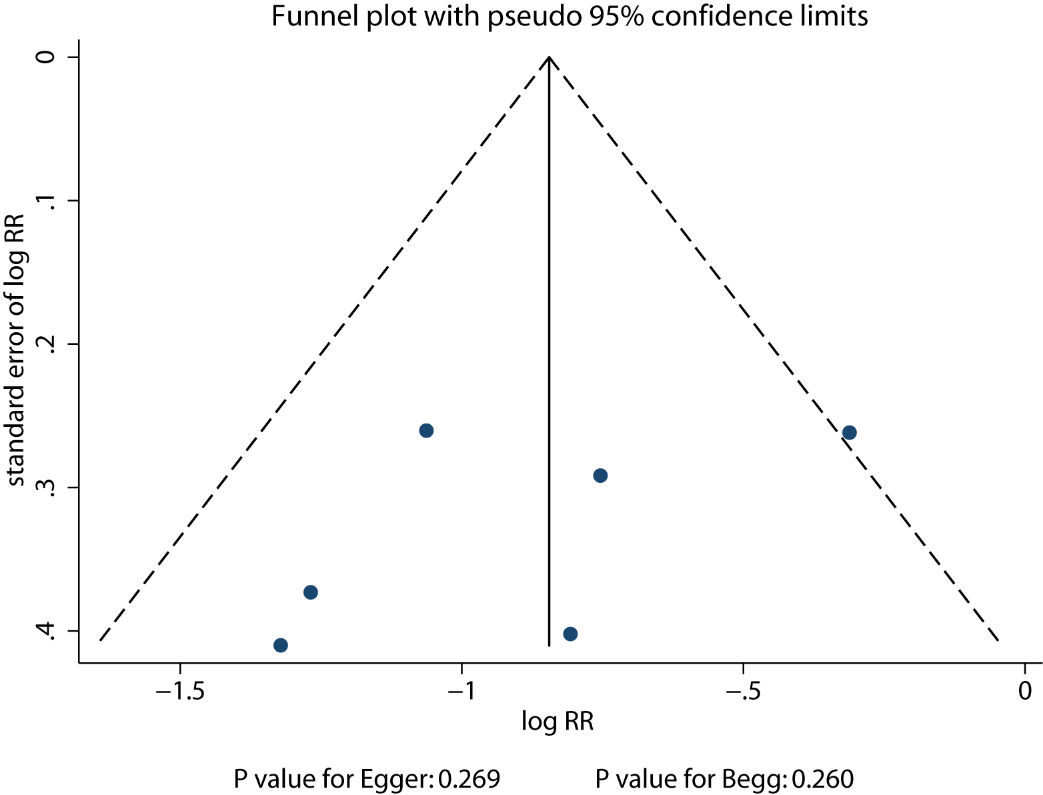


Figure S18. Funnel plot for TLR at 24 months


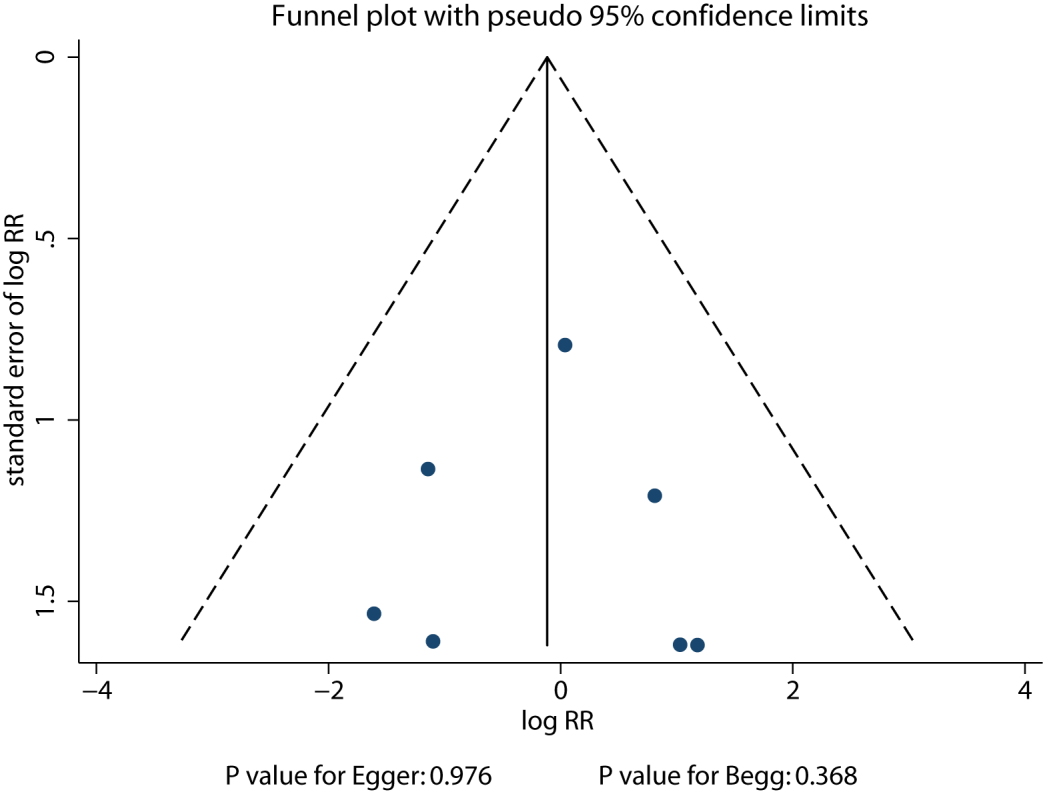


Figure S19. Funnel plot for all-cause mortality at 6 months


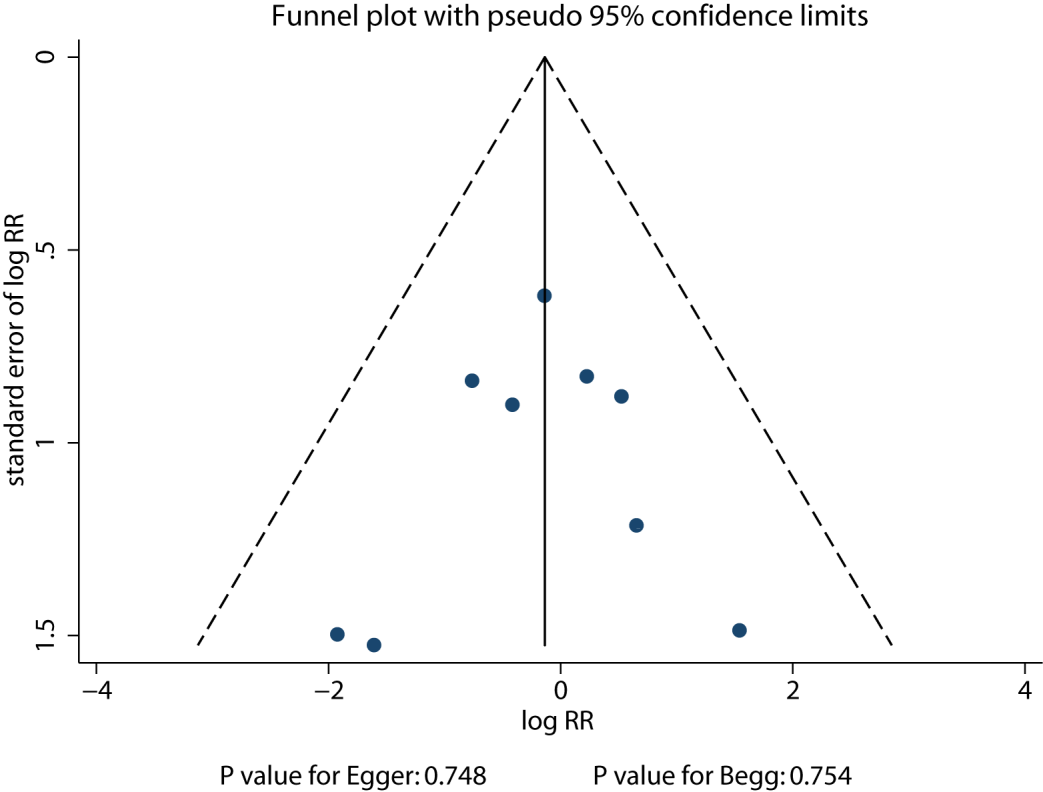


Figure S20. Funnel plot for all-cause mortality at 12 months


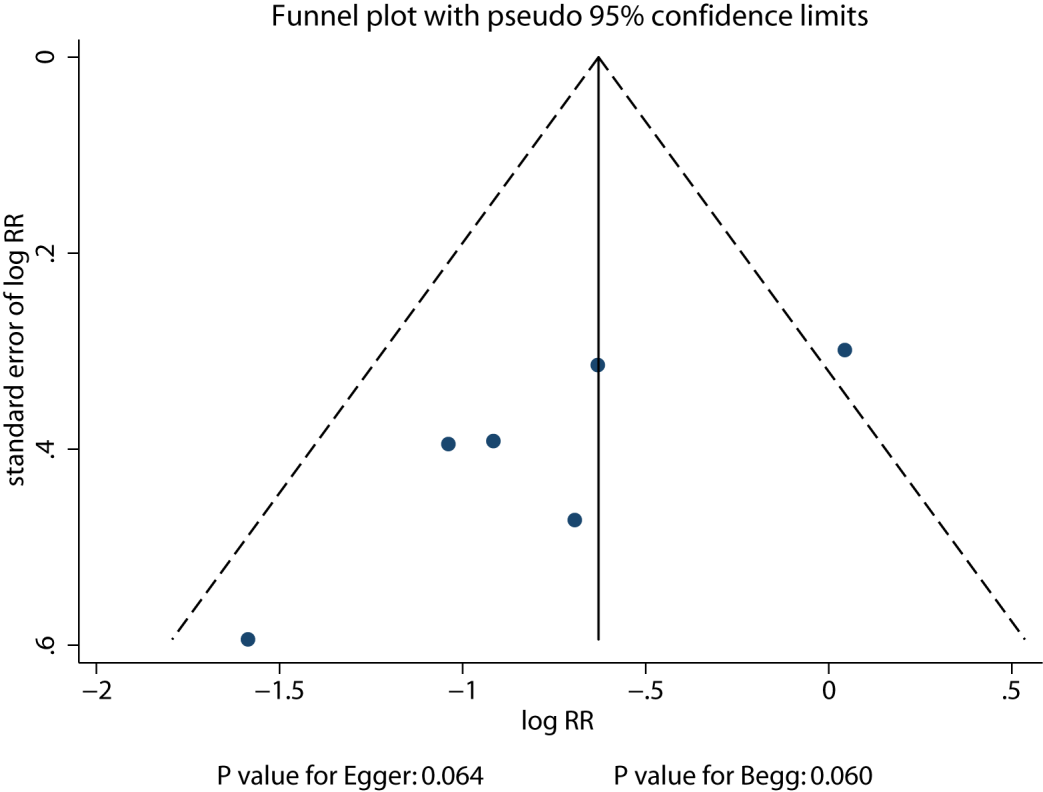


Figure S21. Funnel plot for major adverse events at 12 months


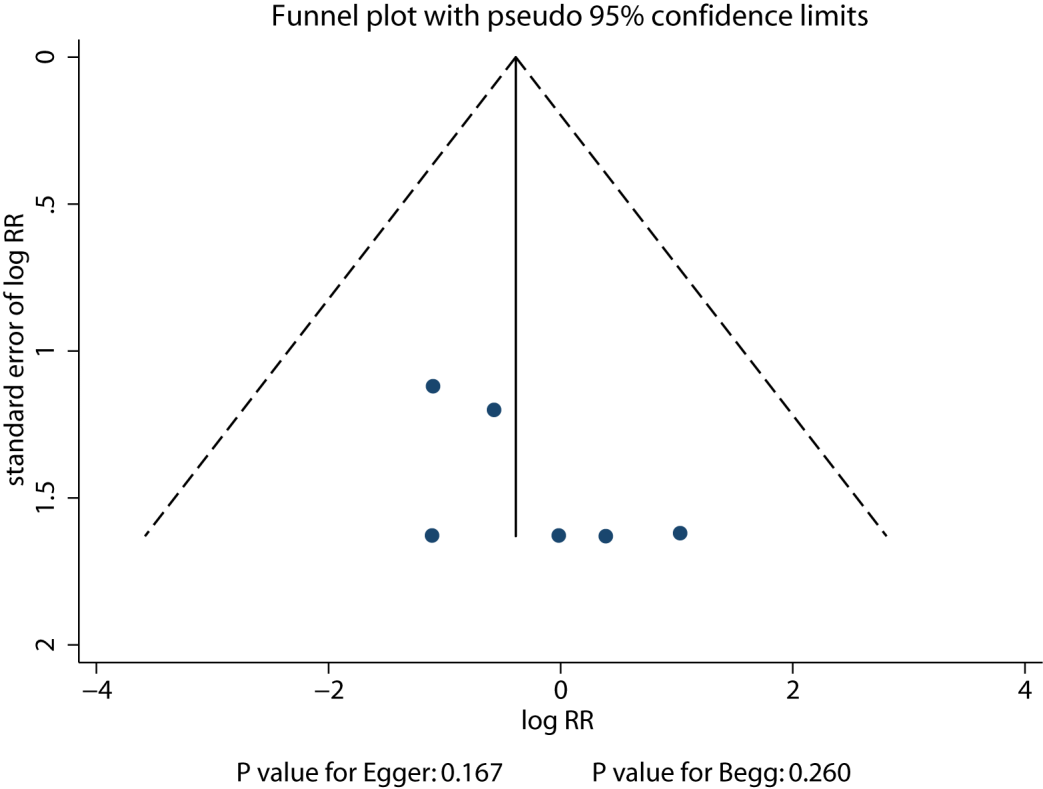


Figure S22. Funnel plot for amputation at 12 months
